# Cross-tissue eQTL enrichment of associations in schizophrenia

**DOI:** 10.1371/journal.pone.0202812

**Published:** 2018-09-06

**Authors:** Francesco Bettella, Andrew A. Brown, Olav B. Smeland, Yunpeng Wang, Aree Witoelar, Alfonso A. Buil Demur, Wesley K. Thompson, Verena Zuber, Anders M. Dale, Srdjan Djurovic, Ole A. Andreassen

**Affiliations:** 1 NORMENT, KG Jebsen Centre for Psychosis Research, University of Oslo, 0424 Oslo, Norway - Division of Mental Health and Addiction, Oslo University Hospital, Oslo, Norway; 2 Department of Genetic Medicine and Development CMU, University of Geneva, Rue Michel-Servet 1, 1211 Geneva, Switzerland; 3 Department of Neurosciences, University of California San Diego, 92093 San Diego, United States of America; 4 Institut for biologisk psykiatri, Psykiatrisk Center Sct. Hans, Boserupvej 2, 4000 Roskilde, Denmark; 5 MRC Biostatistics Unit, Cambridge Institute of Public Health, Robinson Way, CB2 0SR Cambridge, United Kingdom; Universitat de Lleida, SPAIN

## Abstract

The genome-wide association study of the Psychiatric Genomics Consortium identified over one hundred schizophrenia susceptibility loci. The number of non-coding variants discovered suggests that gene regulation could mediate the effect of these variants on disease. Expression quantitative trait loci (eQTLs) contribute to variation in levels of mRNA. Given the co-occurrence of schizophrenia and several traits not involving the central nervous system (CNS), we investigated the enrichment of schizophrenia associations among eQTLs for four non-CNS tissues: adipose tissue, epidermal tissue, lymphoblastoid cells and blood. Significant enrichment was seen in eQTLs of all tissues: adipose (*β* = 0.18, *p* = 8.8 × 10^−06^), epidermal (*β* = 0.12, *p* = 3.1 × 10^−04^), lymphoblastoid (*β* = 0.19, *p* = 6.2 × 10^−08^) and blood (*β* = 0.19, *p* = 6.4 × 10^−06^). For comparison, we looked for enrichment of association with traits of known relevance to one or more of these tissues (body mass index, height, rheumatoid arthritis, systolic blood pressure and type-II diabetes) and found that schizophrenia enrichment was of similar scale to that observed when studying diseases in the context of a more likely causal tissue. To further investigate tissue specificity, we looked for differential enrichment of eQTLs with relevant Roadmap affiliation (enhancers and promoters) and varying distance from the transcription start site. Neither factor significantly contributed to the enrichment, suggesting that this is equally distributed in tissue-specific and cross-tissue regulatory elements. Our analyses suggest that functional correlates of schizophrenia risk are prevalent in non-CNS tissues. This could be because of pleiotropy or the effectiveness of variants affecting expression in different contexts. This suggests the utility of large, single-tissue eQTL experiments to increase eQTL discovery power in the study of schizophrenia, in addition to smaller, multiple-tissue approaches. Our results conform to the notion that schizophrenia is a systemic disorder involving many tissues.

## Introduction

Since the completion of the human genome project, genome-wide association studies (GWAS) have been useful instruments for improving our understanding of the genetics behind human traits. However, the promise of identifying the genetic basis of disease and determine its underlying mechanisms has only marginally been fulfilled.

The schizophrenia GWAS carried out by the Psychiatric Genomics Consortium (PGC) identified many genomic loci plausibly involved in the etiology of the disorder [1] but much of the heritability, estimated from familial studies, is yet to be identified [2, 3]. A considerable portion of the variance in case-control status can be explained in many cohorts using available GWAS data [3, 4], but identifying mechanisms by which these variants act has proved difficult. The approach of GWAS is to independently test a vast genome-wide selection of representative genetic variants for associations with the trait of interest, disregarding any other genetic information. However, it is now known that there are many types of genetic variants with diverse propensities to associate with phenotypic traits [5, 6]. Including functional annotations in the analyses may thus aid in the quest of uncovering the genetic basis of the disease.

As seen by the large numbers of non-coding variants identified by GWASes of complex traits, many causal variants are likely to act through the regulation of a gene rather than through changes in the coding sequence [7–10]. This has boosted the interest in studies of gene expression and has encouraged the development of transcriptomics and proteomics databases and tools [11–18]. Expression quantitative trait loci (eQTLs) are genomic loci involved in variation of mRNA levels. Many eQTLs have been associated with different human phenotypes [8] including schizophrenia [19]. The latter was associated in particular to genetic variations affecting gene expression in various brain tissues [17, 20, 21].

People suffering from schizophrenia are often also affected by disorders of other traits involving non-CNS tissues. Examples include high body mass index (BMI) and obesity, hypertension, cardiovascular disease, as well as disturbances of metabolism and immune system. This has led some to regard schizophrenia as a systemic disease [22]. Recent studies provided support to the hypothesis of common genetic mechanisms underpinning schizophrenia and other disorders [23, 24]. In line with such hypothesis, we looked for association enrichment of eQTLs in non-CNS tissues that may be important for schizophrenia: adipose tissue, epidermal tissue, lymphoblastoid cell lines (LCLs) and whole blood [25]. In order to provide adequate terms of comparison, we also included in the study GWAS data related to other traits and diseases known to be polygenic or relevant to specific non-CNS tissues and thought to share at least part of their pathophysiology with schizophrenia: obesity (high BMI) and type-II diabetes involve metabolic processes known to be often affected in individuals with schizophrenia [26]; hypertension (high blood pressure) is one of the main risk factors for cardiovascular disease which is prevalent among individuals with schizophrenia [27] and may share genetic mechanisms with it [23]; rheumatoid arthritis is a disorder of the immune system which several lines of evidence [1, 24, 28–31] relate to schizophrenia; finally, height is one of the most polygenic traits and is also anticipated to inversely correlate to the propensity to suffer from SCZ [32].

The eQTLs are far from interchangeable but play different roles depending on what tissue they affect expression in and what functional regions of the genome they are situated in [25, 33, 34]. The Roadmap [35] epigenomics project was instrumental for detailing the functional annotation of large portions of the genome and expose the interplay of important regulation mechanisms therein. We investigated the differential enrichment between distal and proximal eQTLs and between eQTLs pertaining to different Roadmap functional annotation categories (see Materials and methods), which could provide additional evidence for tissue specificity.

## Results

### EQTL enrichment in schizophrenia

**Fig 1:**
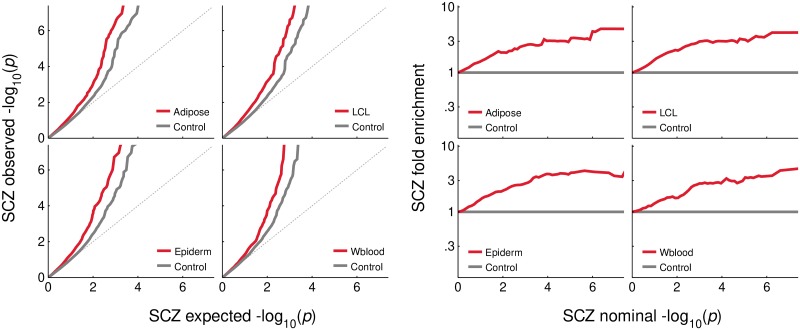
Schizophrenia association enrichment in eQTLs. Q-Q and fold enrichment plots for adipose, epidermal, LCL and whole blood eQTLs. The baseline is determined by respectively matched control SNP sets. The fold enrichment is displayed in logarithmic scale.

The Q-Q and fold enrichment plots suggest a ubiquitous enrichment of associations with schizophrenia (Fig 1) among non-CNS eQTLs that is comparable to the one observed among GTEx brain eQTLs [36] (S1 Fig). In fact, only CommonMind [17] brain eQTLs presented significantly higher schizophrenia association enrichment (S1 Fig, S1 Table) than non-CNS eQTLs, perhaps driven by GTEx-designated cerebellum and possibly hypothalamus eQTLs, although the low number of eQTLs available to the region-specific analyses provides only suggestive evidence in this respect (S2 Fig, S1 Table). The non-CNS eQTL enrichment is reflected in significant association chi-squared test statistics differences (S2 Table) between eQTLs and control variants. However, the deflection in the distribution of the association statistics may be due to factors other than the one we are interested in, i.e. eQTL status. The effects of other such factors were assessed by fitting linear models of the association chi-squared statistics including promoter and enhancer affiliation, and total LD as covariates.

The influence of LD on the linear models was pre-assessed by comparing the LD scores of all eQTL types to those of the respective control variants (S3 Table, S3 and S4 Figs). The eQTLs are ascribed in general more LD than their matched counterparts. Interestingly, this difference seems mostly due to proximal eQTLs (see Materials and methods) active in multiple tissues, since it disappears once these are excluded (data not shown). While potentially intriguing, as it may hint at some selective sweep process, these tests confirm the importance of controlling for LD when assessing enrichment [5].

If an excess of schizophrenia associations exists among eQTLs, the association chi-squared test statistics are expected to be higher for eQTLs than they are for control variants even while covarying with total LD and affiliation to genetic and regulatory categories. The schizophrenia coefficients for tissue-specific eQTLs and control variants, distal and proximal eQTLs, are reported in Table 1. All eQTL types have significantly higher schizophrenia association chi-squared (adipose $\bar{\beta }=0.18$, unadjusted *p* = 8.8*E* − 06; epidermal $\bar{\beta }=0.16$, unadjusted *p* = 3.1*E* − 04; lymphoblastoid $\bar{\beta }=0.2$, unadjusted *p* = 6.2*E* − 08; whole blood $\bar{\beta }=0.19$, unadjusted *p* = 6.4*E* − 06) than the matched control variants. The higher chi-squared are generally reflected in higher estimated proportions of non-null associations *π*_1_ and significantly discern eQTLs from control variants (Mann-Whitney tests). All test statistics are somewhat diminished by exclusion of CommonMind and GTEx brain eQTLs (S4 Table) but do not change in essence.

**Table 1:** Enrichment statistics and general linear model coefficients for squared schizophrenia association z-scores differences between adipose tissue, epidermal tissue, lymphoblastoid cell lines (LCL) and whole blood eQTLs, and matching control variants.

annotation		$\bar{\beta }$	*β* (low 95%)	*β* (high 95%)	*p*	*π*_1_	*p*_MW_
Adipose	eQTL	0.18	0.099	0.25	8.75E-06	0.21	8.03E-12
	control	-0.083	-0.12	-0.042	6.79E-05	0.07	
Epidermal	eQTL	0.12	0.055	0.19	0.00031	0.17	2.18E-06
	control	-0.079	-0.13	-0.033	0.00077	0.076	
LCL	eQTL	0.19	0.12	0.27	6.21E-08	0.14	5.20E-11
	control	-0.097	-0.14	-0.055	4.23E-06	0.082	
Whole blood	eQTL	0.19	0.11	0.27	6.38E-06	0.14	0.0027
	control	-0.0021	-0.045	0.041	0.92	0.098	
All	prox	0.24	0.18	0.30	1.75E-15	0.14	0.80
	dist	0.13	0.083	0.18	5.38E-08	0.19	0.20
	eQTL	0.22	0.18	0.26	5.66E-31	0.17	7.41E-26

$\bar{\beta }$ is the mean effect size over the general linear model replicas with functional genetic affiliation covariates; *p* is the corresponding unadjusted p-value (see methods for more details); *π*_1_ is the estimated proportion of non-null associations; *p*_MW_ is the unadjusted Mann-Whitney test p-value for differences in association chi-squared between eQTL and respective matched control variants; prox stands for proximal eQTLs, dist for distal eQTLs.

To contextualize the observed enrichment, we intersected the TwinsUK eQTLs with LD windows at haplotype *r*^2^ ≥ 0.8 around the 128 LD-independent genomic loci of recognized significance for schizophrenia [1]. The result is reported in Table 2. The overlap corresponds to approximately 12% (15 out of 128) of the loci and is significantly larger than the fraction of independent genomic loci (∼1,000,000) represented by the TwinsUK eQTLs, which we estimate to be between ∼2.8% (OR = 0.24, *p* = 9.5 · 10^−5^) and ∼3.2% (OR = 0.27, *p* = 4.3 · 10^−5^), depending on whether one considers the designated cis-eQTLs (27,974) or an estimate of the number of independent Roadmap promoter loci for the four tissues of interest (∼32,000).

**Table 2:** Cross-tissue eQTLs in the loci with genome-wide significant association with schizophrenia.

Chr	Base pair (GRCh37)	GWAS *p*-value [1]	EQTL	Ensembl gene	HGNC	Tissue
1	8424984	1.17⋅10^−9^	chr1:8464509	ENSG00000142599	RERE	A
	150031490	4.49⋅10^−10^	chr1:149999764	ENSG00000250661	n.a.	B
4	170626552	1.47⋅10^−9^	chr4:170646003	ENSG00000109572	CLCN3	A
5	140143664	4.85⋅10^−8^	chr5:140107679	ENSG00000146007	ZMAT2	L
			chr5:140157427	ENSG00000170445	HARS	A
			chr5:140109155	ENSG00000256453	DND1	E
10	104612335	6.2⋅10^−19^	chr10:104628873	ENSG00000214435	AS3MT	BE
11	57510294	2.24⋅10^−9^	chr11:57424040	ENSG00000156599	ZDHHC5	B
			chr11:57585662	ENSG00000213593	TMX2	L
12	29917265	3.91⋅10^−8^	chr12:29934586	ENSG00000133687	TMTC1	L
	57487814	2.13⋅10^−8^	chr12:57490100	ENSG00000166888	STAT6	E
			chr12:123735937	ENSG00000111325	OGFOD2	AE
			chr12:123689386	ENSG00000111328	CDK2AP1	L
	123665113	1.86⋅10^−14^	chr12:123704844	ENSG00000130921	C12orf65	A
			chr12:123697007			L
			chr12:123689386	ENSG00000235423	n.a.	L
15	40567237	4.18⋅10^−9^	chr15:40569884	ENSG00000137841	PLCB2	B
	91426560	8.3⋅10^−14^	chr15:91426560	ENSG00000140564	FURIN	A
16	29939877	4.55⋅10^−11^	chr16:29924905	ENSG00000149929	HIRIP3	A
	58681393	1.87⋅10^−8^	chr16:58681393	ENSG00000103034	NDRG4	L
19	50091199	4.69⋅10^−8^	chr19:50100295	ENSG00000126460	PRRG2	E
			chr19:50103252	ENSG00000126464	PRR12	A
22	42340844	3.43⋅10^−8^	chr22:42343091	ENSG00000100197	CYP2D6	A

Tissue code: A = adipose, E = epidermal, L = LCL, B = whole blood

The question of whether proximal and distal eQTLs have different incidence of associations with schizophrenia can be addressed with a test in all respects analogous to the one comparing eQTLs to control variants, where proximal and distal eQTLs act as the two categories. In spite of the larger estimated fraction of distal associations, the eQTL’s position relative to the TSS does not make a significant difference: both proximal and distal eQTLs have higher association chi-squared but in similar measure (Table 1).

### Roadmap

**Fig 2:**
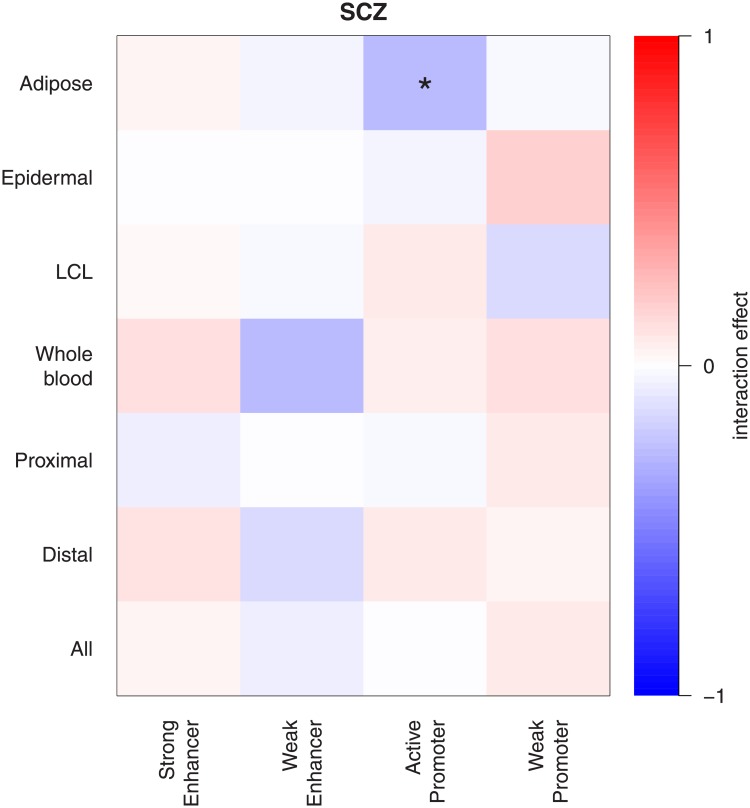
Schizophrenia association enrichment of eQTLs with different Roadmap functional annotations. Chi-squared general linear model coefficients for eQTLs of different tissues (adipose, epidermal, lymphoblastoid cell lines (LCL), whole blood) and location (proximal, distal) affiliated to different Roadmap functional elements. “All” stands for all eQTLs (* *p* < 0.05, ** *p* < 0.001).

The Roadmap functional affiliation effects were estimated upon fitting the linear models described above. These predictably suggest that the variants affiliated to any Roadmap functional elements are considerably more likely to associate to schizophrenia (S5 Table). However, when the linear model fit is restricted to eQTLs and control variants only, the functional affiliation is partially accounted for and the Roadmap functional affiliation association is visibly reduced (S6 Table). Curiously, the reduction is more prominent for enhancers even though these are expected to be farther removed from the coding elements than TwinsUK’s cis-eQTLs. Variations in schizophrenia association propensity between eQTLs acting in different regulatory elements were assessed using the same method. We fitted linear models of schizophrenia association chi-squared test statistics including eQTLxRoadmap “interaction” terms. If the eQTL functional category were of importance the effect size estimates for such terms would be expected to be significantly different from zero. We observed no effect size estimates significantly different from zero, suggesting that if a variant already has an eQTL designation, its placement within diverse genetic functional elements does not to play any important role (Fig 2 and S7 and S8 Tables). The exclusion of CommonMind and GTEx brain eQTls from the analyses did not noticeably alter the results (S9 and S10 Tables).

### Comparison with other phenotypes

**Fig 3:**
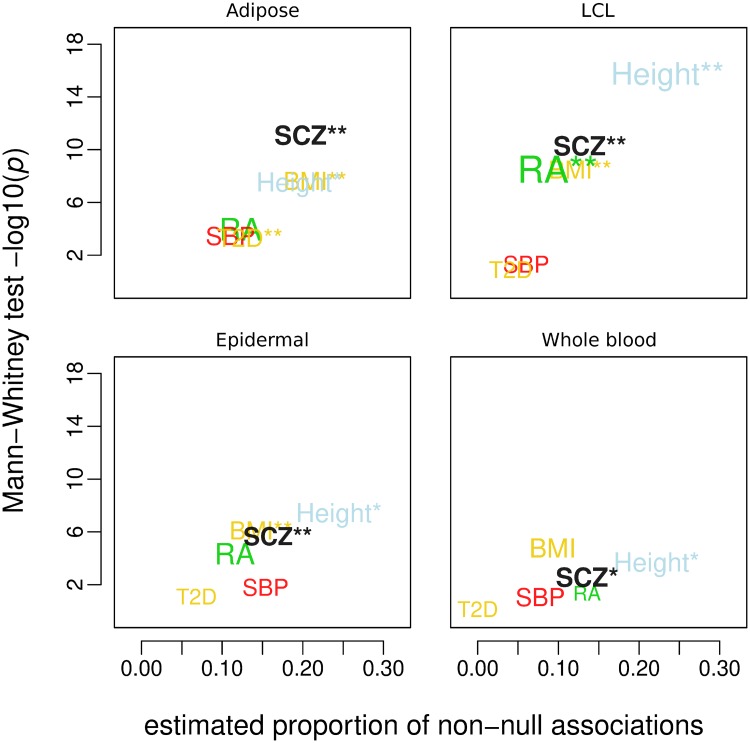
Relationship between polygenicity and eQTL association enrichment across different GWASes. Differences (Mann-Whitney test p-values) in association p-values between eQTLs and control variants of various types as functions of the estimated proportions of non-null associations. The GWAS names or acronyms are color-coded to represent different categories (azure = anthropometric [height]; red = cardiovascular, systolic blood pressure [SBP]; green = immune, rheumatoid arthritis [RA]; gold = metabolic, body mass index [BMI], type-II diabetes [T2D]; black = schizophrenia) and their sizes are proportional to the respective chi-squared linear model coefficients (* *p* < 0.05, ** *p* < 0.001).

The results of the analyses of other GWASes are reported in the supplementary information (S5–S9 Figs, S11–S20 Tables). For comparison with schizophrenia, various measures of eQTL enrichment are reported in Fig 3 (see S10 Fig) for all GWASes. The trait names or acronyms are printed with sizes proportional to the GWAS chi-squared general linear model coefficients. Their coordinates were determined respectively by the estimated proportions of non-null associations (*π*_1_) and the Mann-Whitney −log_10_(*p*)-values for chi-squared differences among eQTLs and control variants. The height GWAS shows widespread eQTL enrichment in concert with a uniformly high estimated proportion of associations. Other GWASes like BMI, RA and SBP show various degrees of enrichment but not always at a significant level. Notably, the schizophrenia GWAS shows as high *π*_1_ as RA, if not higher, among lymphoblastoid eQTLs, and as high *π*_1_ as BMI and height among adipose tissue eQTLs. It must be noted that the various GWASes do not offer the same eQTL coverage. However, we found that the results did not appreciably vary upon restricting the analyses to the minimal common set of GWAS variants (data not shown).

## Discussion

The main finding of the present study is that eQTLs for all investigated tissue types (adipose, epidermal, LCL, blood) are enriched of associations with schizophrenia, suggesting that a part of the risk for the illness involves genetic dysregulation in non-CNS tissue types. The non-CNS eQTL enrichment is comparable to that of GTEx [36] brain eQTLs and only slightly lower than that of CommonMind [17] brain eQTLs and is not driven by any of these.

The observed enrichment is reflected in the intersection between the TwinsUK eQTLs and the genome-wide significant loci identified by the PGC. Instantly recognizable in Table 2 are the experimentally validated eQTLs for the CLCN3 and FURIN genes [17].

As the interaction analyses show, the eQTL prevalence of schizophrenia associations is statistically indifferent to their specific functional annotation. Given the gleaned [25] roles of promoters and enhancers in transcription, respectively context-free and context-specific, this indicates that schizophrenia risk variants are distributed among context-specific and generic transcription functional elements.

Upon comparing different measures of enrichment across different GWASes, we find that the schizophrenia GWAS scores often as high as GWASes of traits for which the respective tissue is known to be of physiological relevance. In adipose tissue eQTLs for instance, the enrichment of schizophrenia associations equals that of BMI and height, while in LCL the estimated proportion of non-null schizophrenia associations is as high as that of non-null RA associations. Such observations may indicate an overlap between the pathophysiological substrate of schizophrenia and that of the other traits, which in turn may be a consequence of genetic pleiotropy, as suggested by earlier findings [23, 24]. A recent study has also posited an “omnigenic” model of genetic risk, and suggested that eQTLs that act in a large number of tissues may have as large a role to play as eQTLs that are tissue specific [37]. The enrichment of associations among non-CNS eQTLs does not go to the detriment of that of eQTLs of brain tissues, the natural substrates for schizophrenia’s pathophysiology. The latter predictably present an even higher incidence of associations with the disorder. The lack of relative enrichment among GTEx brain eQTLs is probably due to their lower mapping power, given the smaller sample they were obtained from. The GTEx consortium reported that brain eQTLs replicated most strongly in other brain tissues, demonstrating that a proportion of the eQTLs mapped are indeed specific to brain tissue, but the low sample size will mean that most tissue specific eQTLs will not be discovered, reducing power to observe enrichment.

The enrichment observed for eQTLs of non-CNS tissues suggests that the genetic makeup of schizophrenia may also involve non-neural tissues. The non significant effect of the eQTL regulatory affiliation corroborates the involvement of specific non-CNS tissues in schizophrenia. This supports the long discussed [28] notion of schizophrenia as a systemic disease [22], and fosters the hypothesis of common genetic mechanisms, in line with proposed pleiotropy such as that with cardiovascular and immune diseases [23, 24]. The findings of the present study complement the ones of several recent studies identifying specific or generic eQTLs associations with schizophrenia in brain tissue [17, 20, 21, 38] in that they extend the search space to non-CNS tissue types. They suggest that schizophrenia risk loci affect a range of human tissues. As hinted by the slight overall effects of promoter and enhancer eQTL affiliation, it is possible for some non-CNS eQTLs to be proxy agents for CNS tissue eQTLs. However, the estimated pairwise overlap between eQTLs pertaining to different tissues is relatively low (∼10%). Although such estimates can be slightly biased by the small sample, they encourage to regard TwinsUK’s eQTLs as quite tissue-specific. This considerations added to the negligible effect of double agents, including those active in the brain, on the analyses (see Materials and methods), suggest that the ones detected here are largely non-CNS effects.

Insofar as they indicate that genetic risk of schizophrenia associates with gene expression across different somatic tissues, our findings could also have clinical implications. At the very least they warrant further research to assess the relevance of altered gene expression for the high somatic co-morbidity associated with schizophrenia, which is a major health concern. In this respect, comprehensive studies of gene expression across the lifespan, such as the Human Brain Transcriptome [14] or the Brainspan [15, 16] studies carried out for brain tissues, would be of considerable interest for non-CNS tissues as well.

A few limitations of the present study should be mentioned. First, some caution may have to be exercised when interpreting the eQTL association enrichment as, due to the intricate patterns of linkage disequilibrium, the overlap of associations with distinct traits could in part be coincidental [39, 40]. Second, the numbers presented here should be regarded in light of the scope of genetic cis-regulation. As highlighted by Buil et al. [25], cis-eQTLs are generally responsible for considerably less than a third and possibly as little as a sixth of the variation in gene expression. Variants affecting trans-regulation have previously been seen to be important in disease [41]. The importance of trans-regulation must therefore be kept in mind when weighing the implications of eQTL enrichment in GWAS. Some trans-regulation will be the downstream consequences of cis-eQTL effects along the genetic pathway, and we would expect to detect enrichment of such signals in our analysis; however, much trans-regulation is expected to be highly tissue-specific, and such enrichment will be detectable only when studying a small minority of tissues. Third, the higher the statistical power the easier it is to discern weak associations. An enrichment of associations is therefore more likely to be detected in the BMI GWAS (sample size *N* = 339, 224) than in the RA GWAS (sample size *N* = 103, 638). The widespread enrichment observed in the schizophrenia GWAS (sample size *N* = 150, 064) may be due in part to its relatively high statistical power. Its cross-tissue enrichment pattern, however, resembles the ones observed for BMI and height, two highly polygenic traits, and is quite different from the one observed for the T2D GWAS (sample size *N* = 149, 821), a roughly equally powered study. We therefore conclude that the different polygenic architectures must also be a relevant factor.

In summary, we find that investigating functional correlates of schizophrenia risk loci in non-CNS tissues may be productive. The observed non-CNS enrichment of schizophrenia association could be due to pleiotropic effects or increased effectiveness of variants that work in many different environmental contexts. To further delineate the functional molecular mechanisms underlying schizophrenia, it could therefore be useful to complement multiple tissue eQTL experiments with larger single tissue eQTL experiments.

## Materials and methods

### Material

#### GWAS summary statistics

We used summary *p*-value statistics from schizophrenia (SCZ, *N* = 150, 064) [1] as well as body mass index (BMI, *N* = 339, 224) [42], height (*N* = 253, 288) [43], rheumatoid arthritis (RA, *N* = 103, 638) [44], systolic blood pressure (SBP, *N* = 203, 056) [45] and type-II diabetes (T2D, *N* = 149, 821) [46] GWASes. Two sets of about 2.5 and 9 million SNPs encompassing high-quality variants from all GWASes [5], were used for better comparisons in analyses involving non-CNS eQTLs only, and brain as well as non-CNS eQTLs, respectively.

#### TwinsUK eQTLs

The eQTL data were obtained from the TwinsUK sample [47] comprising 856 Caucasian female individuals. We regard these to be suitable due to the size of the TwinsUK sample and because the sample was collected from healthy living individuals and thus free of post-mortem biases. As previously reported [25], TwinsUK’s eQTLs are represented by the single nucleotide variants with strongest association within each locus, with cis-gene expression in adipose tissue, epidermal tissue, lymphoblastoid cell lines (LCLs), and whole blood. The variants used in the current study (9166 adipose tissue, 8731 epidermal tissue, 9551 LCLs, and 5313 blood eQTLs; see [25] for a full description of the eQTL detection method) were designated as eQTLs at a 1% FDR level. Due to their location and the observed association, eQTLs have properties which can differ from other variants in the genome, including minor allele frequency, and regional conservation score and linkage disequilibrium structure, which may cause them to show enrichment in GWAS independently of their role in genetic regulation. To control for these factors when looking for enrichment, we used a set of 40,194 “control” eQTLs produced in [25], which were matched with the eQTLs on two proxies for these factors (minor allele frequency and distance to TSS). In addition to the criteria used in [25] we required that the control eQTLs did not present significant evidence of association with the expression levels of any genes (*p*-value ≥ 10^−4^). A census of the eQTLs included in the analyzed data sets is reported in S21 Table. As described by Buil et al. [25], gene regulation is not always tissue-specific and some loci act as eQTLs in more than one type of tissue. The pairwise overlap counts are shown in S11 Table. All analyses described below were repeated after exclusion of double agents and found to be robust to such exclusion.

#### Brain eQTLs

GTEx version 7 [36] and CommonMind release 1.2 [17] eQTL data were downloaded from the projects’ respective web-services (www.gtexportal.org, www.synapse.org). A wealth of region-specific (amygdala, anterior cingulate cortex, caudate basal ganglia, cerebellar hemisphere, cerebellum, cortex, frontal cortex, hippocampus, hypothalamus, nucleus accumbens basal ganglia, putamen basal ganglia, spinal cord cervical, substantia nigra) brain eQTLs at FDR < 0.01 were readily available in the GTEx data, allowing in detail as well as whole brain analyses. GTEx brain eQTLs were defined as eQTLs for any of the GTEx brain regions. The CommonMind eQTLs were from a larger set of dorsolateral prefrontal cortex tissue samples. The CommonMind brain eQTLs resulted from the intersection of the two eQTL sets designated respectively in the analysis including and the one not including surrogate variables. GTEx and CommonMind eQTLs at the same FDR level were subsequently intersected to obtain a consensus set of brain eQTLs.

#### Roadmap annotation

Even if all eQTLs considered here are cis-eQTLs, some differences may still exist between eQTLs located at different positions with respect to promoters. We therefore subdivided eQTLs into proximal and distal eQTLs, depending on their distance from the TSS. We set the threshold at 25 kbp to equally populate the two categories. In order to further detail the eventual enrichment patterns, eQTLs were further assigned one of four tissue-specific Roadmap functional annotations: strong enhancer, weak enhancer, active promoter, and weak promoter eQTLs. The functional annotations were extracted from adipose nuclei tracks (E063) for adipose tissue eQTLs, from normal human epidermal keratinocytes tracks (E127) for epidermal tissue eQTLs, from lymphoblastoid cell lines tracks (E116) for lymphoblastoid cell lines eQTLs, and from primary mononuclear cells tracks (E062) for whole blood eQTLs. The distribution of eQTLs across all functional elements is reported in S22 Table.

### Statistics

#### Random linkage disequilibrium-based pruning

The TwinsUK eQTLs are not guaranteed to be independent, because different genes can have different eQTLs in linkage disequilibrium with one another. A possible way to account for the known intricate correlation structure of the genome data is to include linkage disequilibrium (LD) as an integral part of the analyses [48]. Another way is to reduce the bias caused by LD by restricting the analyses to near-independent variants. When pursuing the second avenue, the problem of choosing LD-independent representatives arises. To reduce sampling bias, we generated ten sets of near-independent variants (LD *r*^2^ < 0.2 within 1Mbase) picked at random [49] with no replacement to repeatedly perform our analyses on. In order for the genomic correction to be representative of these sets of variants, the genome-wide intergenic correction factor [5] was computed across such sets (median of medians) and applied to all GWAS summary statistics before the ensuing analyses. All results reported are robust to the choice of near-independent variants sets.

#### Conditional fold enrichment and quantile-quantile plots

It is common practice to use quantile-quantile (Q-Q) plots to compare two distributions; Q-Q plots are used in GWAS to compare the expected −log_10_(*p*)-value distribution to the one actually observed and thus assess the non-spurious association content of the latter. Further leftward deflections of the observed distribution in a subset of interest reflect a higher incidence, in other words, enrichment, of low *p*-values (high −log_10_(*p*)-values) in such subset.

Fold enrichment plots are also used to visualize enrichment as a function of the association *p*-value [50]. They were obtained here by comparing the empirical cumulative distributions of −log_10_(*p*)-values for SNP association among eQTLs and control variants. Each eQTL subset S’s fold enrichment was calculated as the cumulative distribution functions (CDFs) ratio $CDF_{S}/CDF_{\text{control}}$ between the −log_10_(*p*) cumulative distribution for ${\text{eQTL}}_{S}$ and the −log_10_(*p*) cumulative distribution for the respective control variants. Left-cumulative association −log_10_(*p*)-value bin centers are reported on the x-axis, fold enrichment on the y-axis [51].

#### Chi-squared general linear models and enrichment estimates

The fold enrichment plots are effective in conveying a visual impression of enrichment. However, they do not provide any quantitative measure of enrichment and are not suited to control for potential mediating effects. General linear models allow to account for any such effects and, assuming no strong interdependence among predictor variables, to assess their significance. To get quantities with better statistical properties, we converted the association *p*-values to z-scores and squared these (*z*^2^ = *F*^−1^(*p*/2)^2^, where *F* is the standard normal cumulative distribution function) to recover the original association chi-squared test statistic. We then fitted a chi-squared general linear model [5] including eQTL and Roadmap categories [35], and total LD score [48] as covariates. Interactions between eQTL and Roadmap or genic categories were subsequently included to detail the effect of the eQTLs’ affiliation to specific functional elements. The reported effect sizes and *p*-values are meta-analysis effect sizes and *p*-values across the ten analyses relative to the single pruned sets of eQTL and control variants. In order to provide a full characterization of the enrichment, we also compared the association chi-squared among different eQTL types and corresponding matched control variants (Mann-Whitney tests) and estimated the proportion *π*_1_ of non-null associations in the two groups using R’s qvalue package [52, 53]. The Mann-Whitney tests and the *π*_1_ estimations were performed on the full set of variants.

## Supporting information

S1 Fig Enrichment plots for CommonMind, GTEx, GTEx/CommonMind consensus brain and non-CNS eQTLs. The top panels contain Q-Q and fold enrichment plots for the CommonMind, GTEx brain and TwinsUK non-CNS eQTLs. The bottom panels contain relative fold enrichment plots for CommonMind and GTEx and for GTEx/CommonMind consensus brain eQTLs compared to non-CNS eQTL variants.
(PDF)

S2 Fig Enrichment plots for GTEx/CommonMind consensus region-specific brain eQTLs. The fold enrichment is relative to non-CNS eQTL variants.
(PDF)

S3 Fig Histograms of total LD. Total LD-tagging power of eQTLs and control SNPs, and proximal (prox) and distal (dist) eQTLs.
(PDF)

S4 Fig Total LD for different eQTL types and control variants in the study. Box plots are overlaid on kernel density plots [54].
(PDF)

S5 Fig BMI association enrichment in eQTLs. Q-Q and fold enrichment plots for adipose, epidermal, LCL and whole blood eQTLs. The baseline is determined by respectively matched control SNP sets. The fold enrichment is displayed in logarithmic scale.
(PDF)

S6 Fig Height association enrichment in eQTLs. Q-Q and fold enrichment plots for adipose, epidermal, LCL and whole blood eQTLs. The baseline is determined by respectively matched control SNP sets. The fold enrichment is displayed in logarithmic scale.
(PDF)

S7 Fig Rheumatoid arthritis association enrichment in eQTLs. Q-Q and fold enrichment plots for adipose, epidermal, LCL and whole blood eQTLs. The baseline is determined by respectively matched control SNP sets. The fold enrichment is displayed in logarithmic scale.
(PDF)

S8 Fig Systolic blood pressure association enrichment in eQTLs. Q-Q and fold enrichment plots for adipose, epidermal, LCL and whole blood eQTLs. The baseline is determined by respectively matched control SNP sets. The fold enrichment is displayed in logarithmic scale.
(PDF)

S9 Fig Type-II diabetes association enrichment in eQTLs. Q-Q and fold enrichment plots for adipose, epidermal, LCL and whole blood eQTLs. The baseline is determined by respectively matched control SNP sets. The fold enrichment is displayed in logarithmic scale.
(PDF)

S10 Fig Differences (Mann-Whitney test p-values) in association p-values between proximal and distal eQTLs as functions of the estimated proportions of non-null associations. The GWAS names or acronyms are color-coded to represent different categories (azure = anthropometric, [height]; red = cardiovascular, systolic blood pressure [SBP]; green = immune, rheumatoid arthritis [RA]; gold = metabolic, body mass index [BMI], type-II diabetes [T2D]; black = schizophrenia) and their sizes are proportional to the respective ANCOVA coefficients (* *p* < 0.05, ** *p* < 0.001).
(PDF)

S11 Fig eQTL distribution across tissues. As established in [25], eQTLs have some tendency to act in more than one tissue.
(PDF)

S1 Table Schizophrenia association chi-squared general linear model coefficients for GTEx brain and GTEx/CommonMind consensus brain eQTLs compared to non-CNS eQTLs. The test statistics refer to a general linear model of all brain and non-CNS variants in the ∼9 million variant template.
(PDF)

S2 Table Schizophrenia association chi-squared differences between various eQTL types and matching control variants. (PDF)

S3 Table Total LD differences between various eQTL types and matching control variants. (PDF)

S4 Table Enrichment statistics and general linear model coefficients for squared schizophrenia association z-scores differences between adipose tissue, epidermal tissue, lymphoblastoid cell lines (LCL) and whole blood eQTLs, and matching control variants. All eQTLs designated by CommonMind or GTEx as brain eQTLs were excluded from these analyses.
(PDF)

S5 Table Schizophrenia association chi-squared general linear model coefficients for the four Roadmap functional affiliations. (PDF)

S6 Table Schizophrenia association chi-squared general linear model coefficients for the four Roadmap functional affiliations restricted to eQTLs and control variants. (PDF)

S7 Table Schizophrenia association chi-squared general linear model coefficients for tissue-specific eQTLs with different functional affiliations. The test statistics refer to the respective interaction terms. The interaction with TotLD represents the enrichment ascribable to the eQTLs irrespective of their LD-tagging power. Enhancer and Promoter affiliations were assigned by Roadmap in the corresponding tissues.
(PDF)

S8 Table Schizophrenia association chi-squared general linear model coefficients for all, proximal or distal eQTLs with different functional affiliations. The test statistics refer to the respective interaction terms. The interaction with TotLD represents the enrichment ascribable to the eQTLs irrespective of their LD-tagging power. Enhancer and Promoter affiliations were assigned by Roadmap in the corresponding tissues.
(PDF)

S9 Table Schizophrenia association chi-squared general linear model coefficients for tissue-specific eQTLs with different functional affiliations upon exclusion of CommonMind and GTEx brain eQTLs. The test statistics refer to the respective interaction terms. The interaction with TotLD represents the enrichment ascribable to the eQTLs irrespective of their LD-tagging power. Enhancer and Promoter affiliations were assigned by Roadmap in the corresponding tissues.
(PDF)

S10 Table Schizophrenia association chi-squared general linear model coefficients for all, proximal or distal eQTLs with different functional affiliations upon exclusion of CommonMind and GTEx brain eQTLs. The test statistics refer to the respective interaction terms. The interaction with TotLD represents the enrichment ascribable to the eQTLs irrespective of their LD-tagging power. Enhancer and Promoter affiliations were assigned by Roadmap in the corresponding tissues.
(PDF)

S11 Table Enrichment statistics and general linear model coefficients for squared BMI association z-scores differences between adipose tissue, epidermal tissue, lymphoblastoid cell lines (LCL) and whole blood eQTLs, and matching control variants. (PDF)

S12 Table BMI association chi-squared general linear model coefficients for all eQTL types with the four Roadmap functional affiliations. Enhancer and Promoter affiliations were assigned by Roadmap in the corresponding tissues.
(PDF)

S13 Table Enrichment statistics and general linear model coefficients for squared Height association z-scores differences between adipose tissue, epidermal tissue, lymphoblastoid cell lines (LCL) and whole blood eQTLs, and matching control variants. (PDF)

S14 Table Height association chi-squared general linear model coefficients for all eQTL types with the four Roadmap functional affiliations. Enhancer and Promoter affiliations were assigned by Roadmap in the corresponding tissues.
(PDF)

S15 Table Enrichment statistics and general linear model coefficients for squared RA association z-scores differences between adipose tissue, epidermal tissue, lymphoblastoid cell lines (LCL) and whole blood eQTLs, and matching control variants. (PDF)

S16 Table Rheumatoid arthritis association chi-squared general linear model coefficients for all eQTL types with the four Roadmap functional affiliations. Enhancer and Promoter affiliations were assigned by Roadmap in the corresponding tissues.
(PDF)

S17 Table Enrichment statistics and general linear model coefficients for squared SBP association z-scores differences between adipose tissue, epidermal tissue, lymphoblastoid cell lines (LCL) and whole blood eQTLs, and matching control variants. (PDF)

S18 Table Systolic blood pressure association chi-squared general linear model coefficients for all eQTL types with the four Roadmap functional affiliations. Enhancer and Promoter affiliations were assigned by Roadmap in the corresponding tissues.
(PDF)

S19 Table Enrichment statistics and general linear model coefficients for squared T2D association z-scores differences between adipose tissue, epidermal tissue, lymphoblastoid cell lines (LCL) and whole blood eQTLs, and matching control variants. (PDF)

S20 Table Type-II diabetes association chi-squared general linear model coefficients for all eQTL types with the four Roadmap functional affiliations. Enhancer and Promoter affiliations were assigned by Roadmap in the corresponding tissues.
(PDF)

S21 Table eQTLs and matched control variants census in the data set used. The eQTLs and control variants from [25] were projected onto templates of ∼2.5 million variants (∼9 million variants for analyses involving brain eQTLs) with known pairwise LD.
(PDF)

S22 Table eQTL VS ENCODE demographics. The numbers refer to the ∼9 million template.
(PDF)
