# Supplementary material for: Cross-tissue eQTL enrichment of associations in schizophrenia
Source: PLoS One. 2018 Sep 6;13(9):e0202812. doi: 10.1371/journal.pone.0202812 (PMC6126834; doi:10.1371/journal.pone.0202812)
Supplement: S1 Fig — The top panels contain Q-Q and fold enrichment plots for the CommonMind, GTEx brain and TwinsUK non-CNS eQTLs. The bottom panels contain relative fold enrichment plots for CommonMind and GTEx and for GTEx/CommonMind consensus brain eQTLs compared to non-CNS eQTL variants. (PDF) [file pone.0202812.s001.pdf]

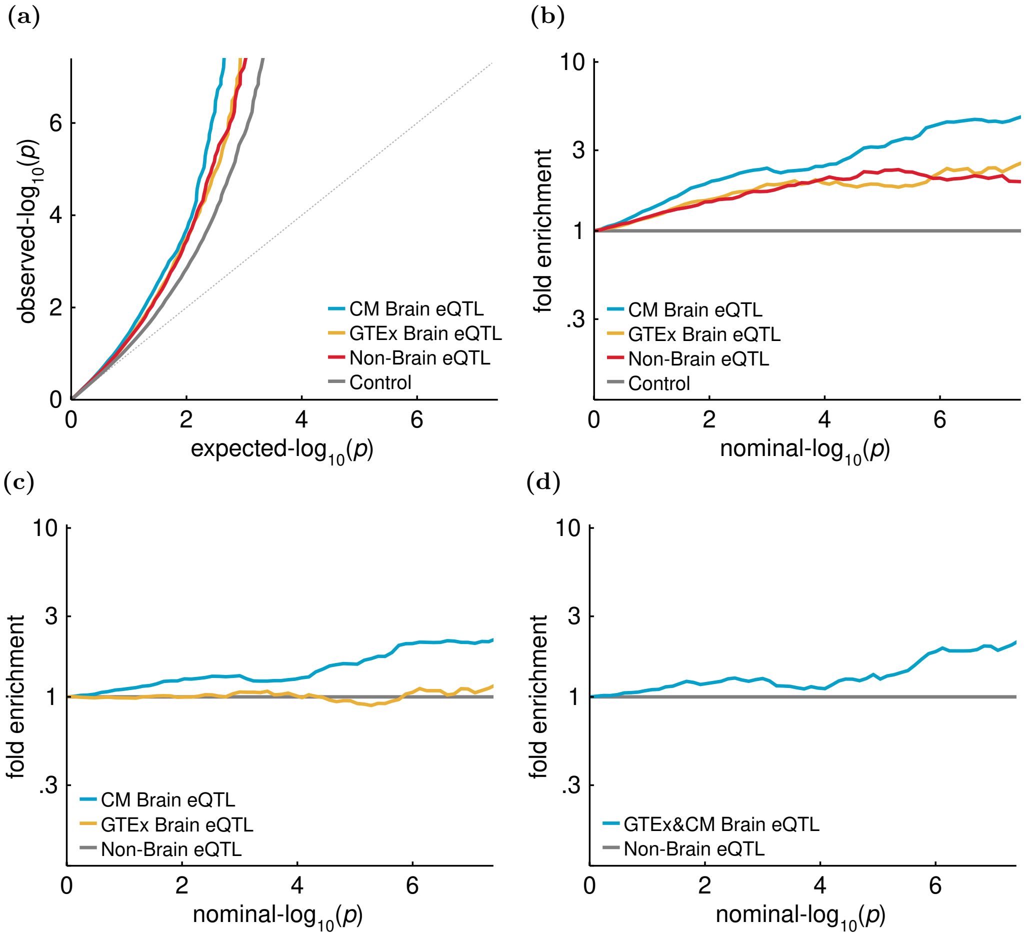

S1 Fig Enrichment plots for CommonMind, GTEx, GTEx/CommonMind consensus brain and non-CNS eQTLs. The top panels contain Q-Q and fold enrichment plots for the CommonMind, GTEx brain and TwinsUK non-CNS eQTLs. The bottom panels contain relative fold enrichment plots for CommonMind and GTEx and for GTEx/CommonMind consensus brain eQTLs compared to non-CNS eQTL variants.
