# Supplementary material for: Cross-tissue eQTL enrichment of associations in schizophrenia
Source: PLoS One. 2018 Sep 6;13(9):e0202812. doi: 10.1371/journal.pone.0202812 (PMC6126834; doi:10.1371/journal.pone.0202812)
Supplement: S2 Fig — The fold enrichment is relative to non-CNS eQTL variants. (PDF) [file pone.0202812.s002.pdf]

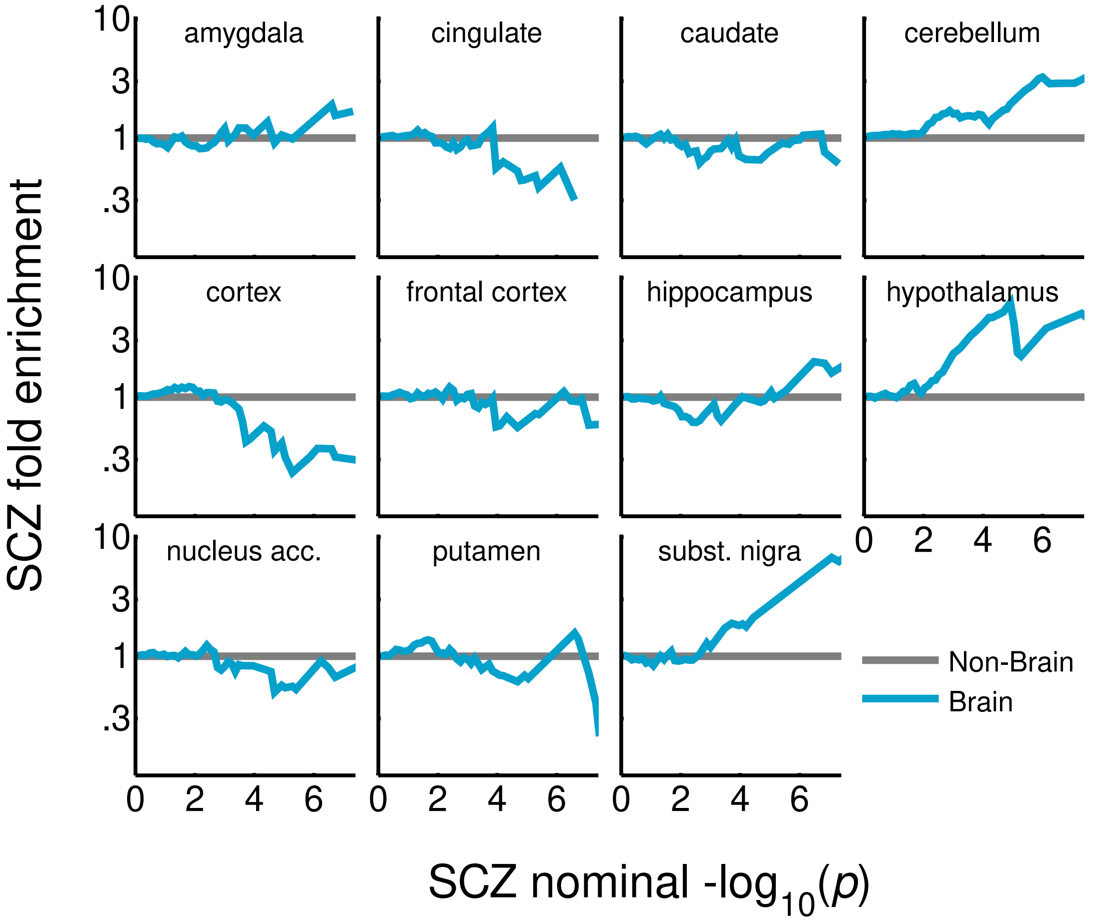

S2 Fig Enrichment plots for GTEx/CommonMind consensus region-specific brain eQTLs. The fold enrichment is relative to non-CNS eQTL variants.
