# Supplementary material for: Cross-tissue eQTL enrichment of associations in schizophrenia
Source: PLoS One. 2018 Sep 6;13(9):e0202812. doi: 10.1371/journal.pone.0202812 (PMC6126834; doi:10.1371/journal.pone.0202812)
Supplement: S3 Fig — Total LD-tagging power of eQTLs and control SNPs, and proximal (prox) and distal (dist) eQTLs. (PDF) [file pone.0202812.s003.pdf]

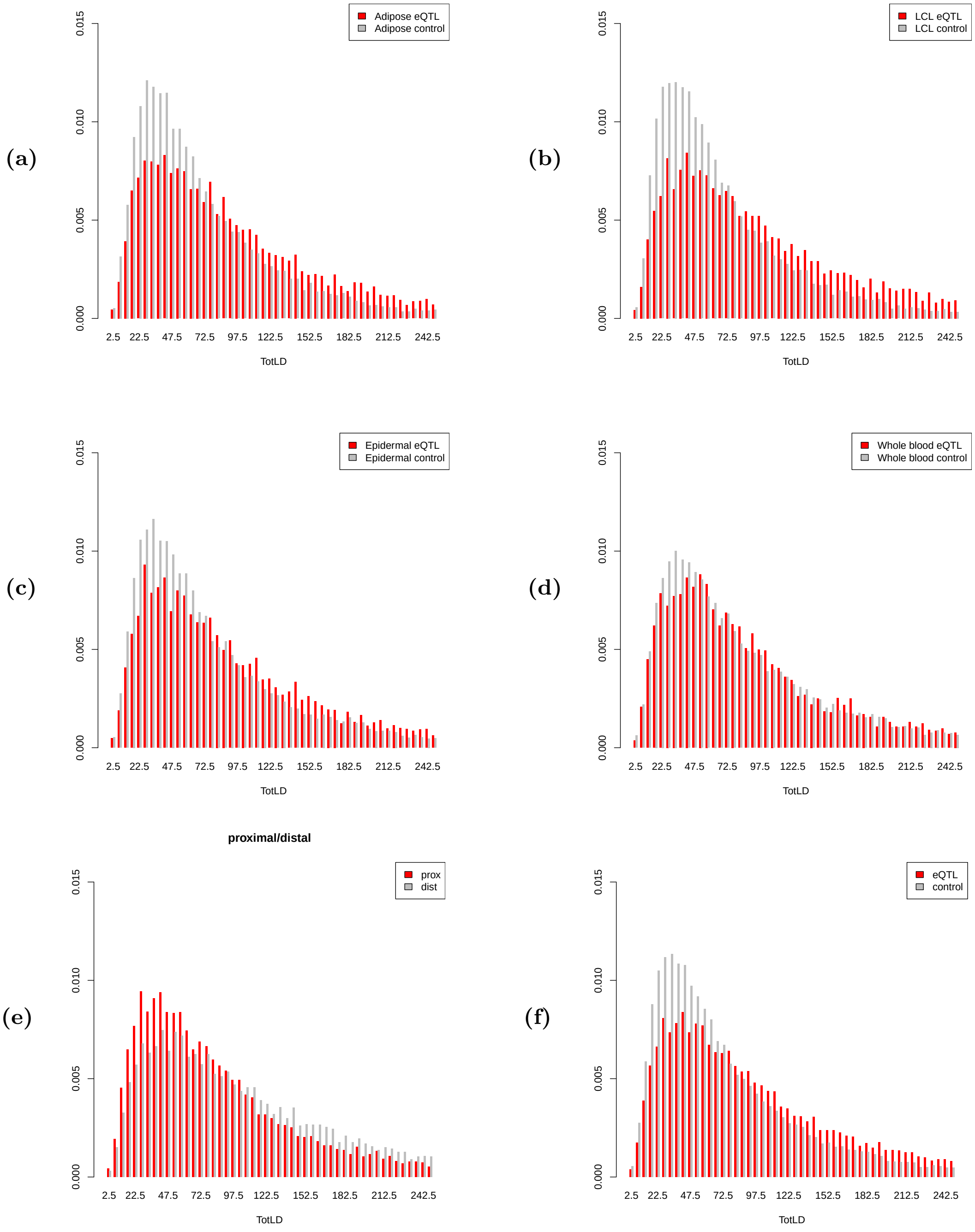

S3 Fig Histograms of total LD. Total LD-tagging power of eQTLs and control SNPs, and proximal (prox) and distal (dist) eQTLs.
