# Supplementary material for: Cross-tissue eQTL enrichment of associations in schizophrenia
Source: PLoS One. 2018 Sep 6;13(9):e0202812. doi: 10.1371/journal.pone.0202812 (PMC6126834; doi:10.1371/journal.pone.0202812)
Supplement: S4 Fig — Box plots are overlaid on kernel density plots [54]. (PDF) [file pone.0202812.s004.pdf]

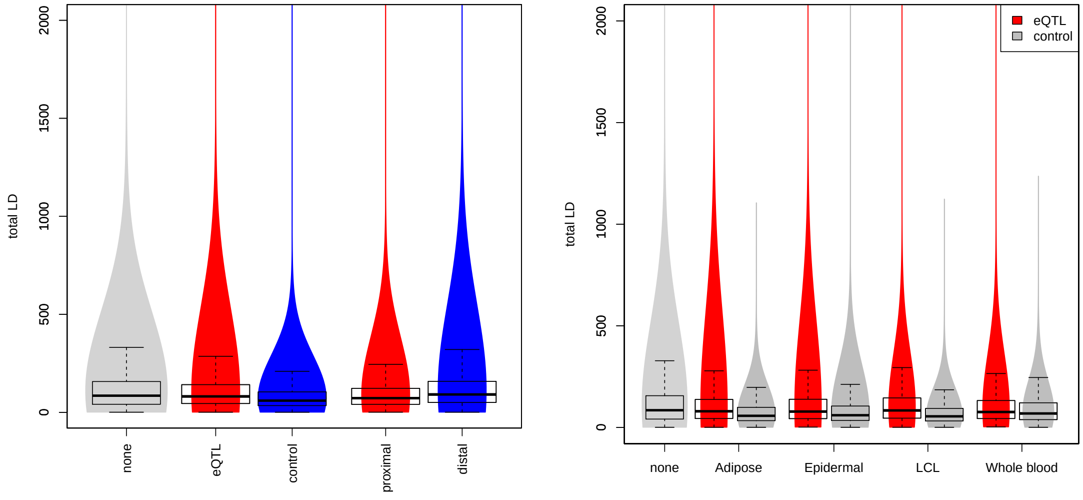

(a) (b)

S4 Fig Total LD for different eQTL types and control variants in the study. Box plots are overlaid on kernel density plots [1].

1. Hintze JL, Nelson RD. Violin plots: a box plot-density trace synergism. The American Statistician. 1998;52(2):181–184.
