# Supplementary material for: Cross-tissue eQTL enrichment of associations in schizophrenia
Source: PLoS One. 2018 Sep 6;13(9):e0202812. doi: 10.1371/journal.pone.0202812 (PMC6126834; doi:10.1371/journal.pone.0202812)
Supplement: S8 Fig — Q-Q and fold enrichment plots for adipose, epidermal, LCL and whole blood eQTLs. The baseline is determined by respectively matched control SNP sets. The fold enrichment is displayed in logarithmic scale. (PDF) [file pone.0202812.s008.pdf]

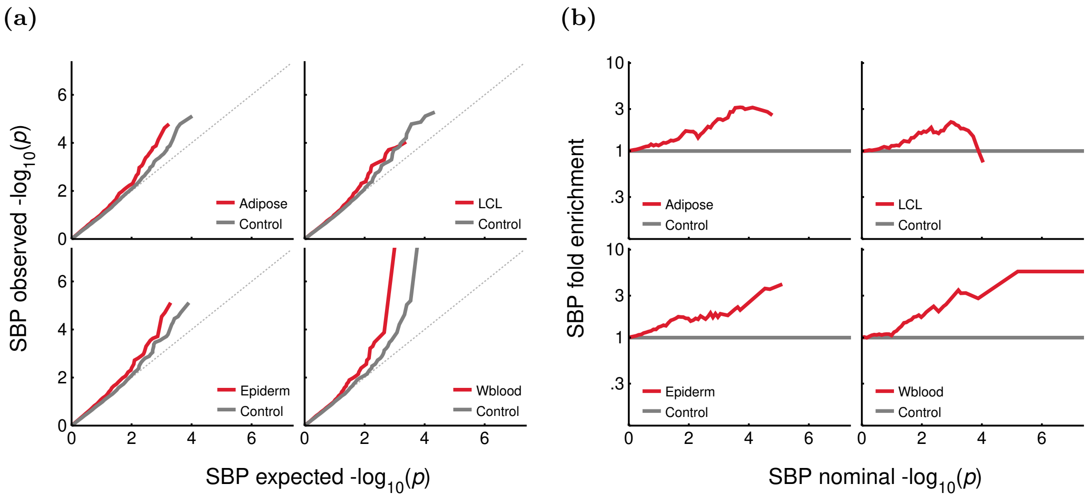

S8 Fig Systolic blood pressure association enrichment in eQTLs. Q-Q and fold enrichment plots for adipose, epidermal, LCL and whole blood eQTLs. The baseline is determined by respectively matched control SNP sets. The fold enrichment is displayed in logarithmic scale.
