# Supplementary material for: Cross-tissue eQTL enrichment of associations in schizophrenia
Source: PLoS One. 2018 Sep 6;13(9):e0202812. doi: 10.1371/journal.pone.0202812 (PMC6126834; doi:10.1371/journal.pone.0202812)
Supplement: S10 Fig — The GWAS names or acronyms are color-coded to represent different categories (azure = anthropometric, [height]; red = cardiovascular, systolic blood pressure [SBP]; green = immune, rheumatoid arthritis [RA]; gold = metabolic, body mass index [BMI], type-II diabetes [T2D]; black = schizophrenia) and their sizes are proportional to the respective ANCOVA coefficients (* p < 0.05, ** p < 0.001). (PDF) [file pone.0202812.s010.pdf]

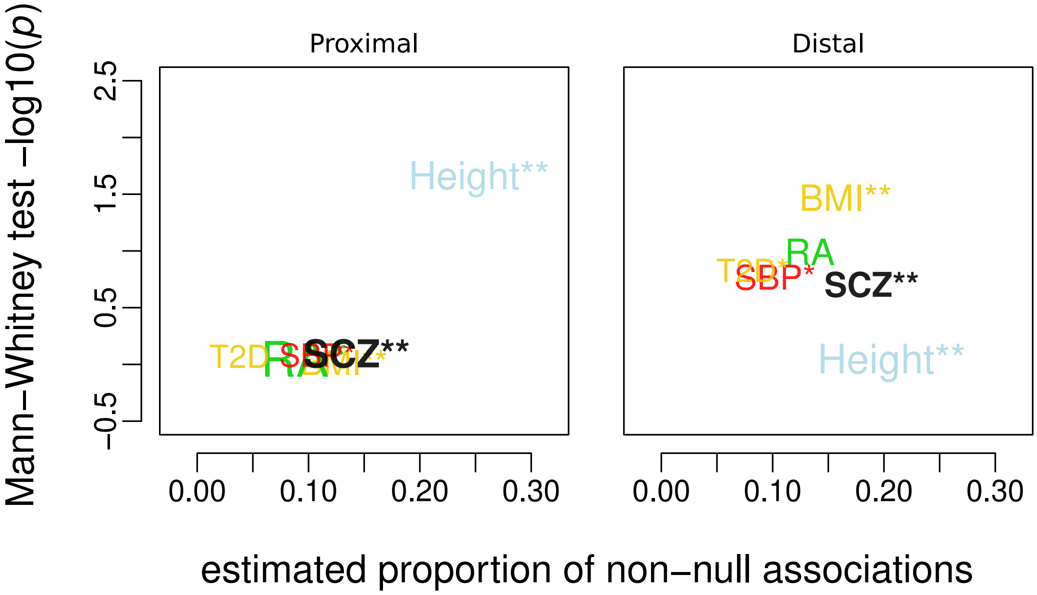

S10 Fig Differences (Mann-Whitney test p-values) in association p-values between proximal and distal eQTLs as functions of the estimated proportions of non-null associations. The GWAS names or acronyms are color-coded to represent different categories (azure=anthropometric, [height]; red=cardiovascular, systolic blood pressure [SBP]; green=immune, rheumatoid arthritis [RA]; gold=metabolic, body mass index [BMI], type-II diabetes [T2D]; black=schizophrenia) and their sizes are proportional to the respective ANCOVA coefficients (* $p < 0.05$, ** $p < 0.001$).
