# Supplementary material for: Cross-tissue eQTL enrichment of associations in schizophrenia
Source: PLoS One. 2018 Sep 6;13(9):e0202812. doi: 10.1371/journal.pone.0202812 (PMC6126834; doi:10.1371/journal.pone.0202812)
Supplement: S11 Fig — As established in [25], eQTLs have some tendency to act in more than one tissue. (PDF) [file pone.0202812.s011.pdf]

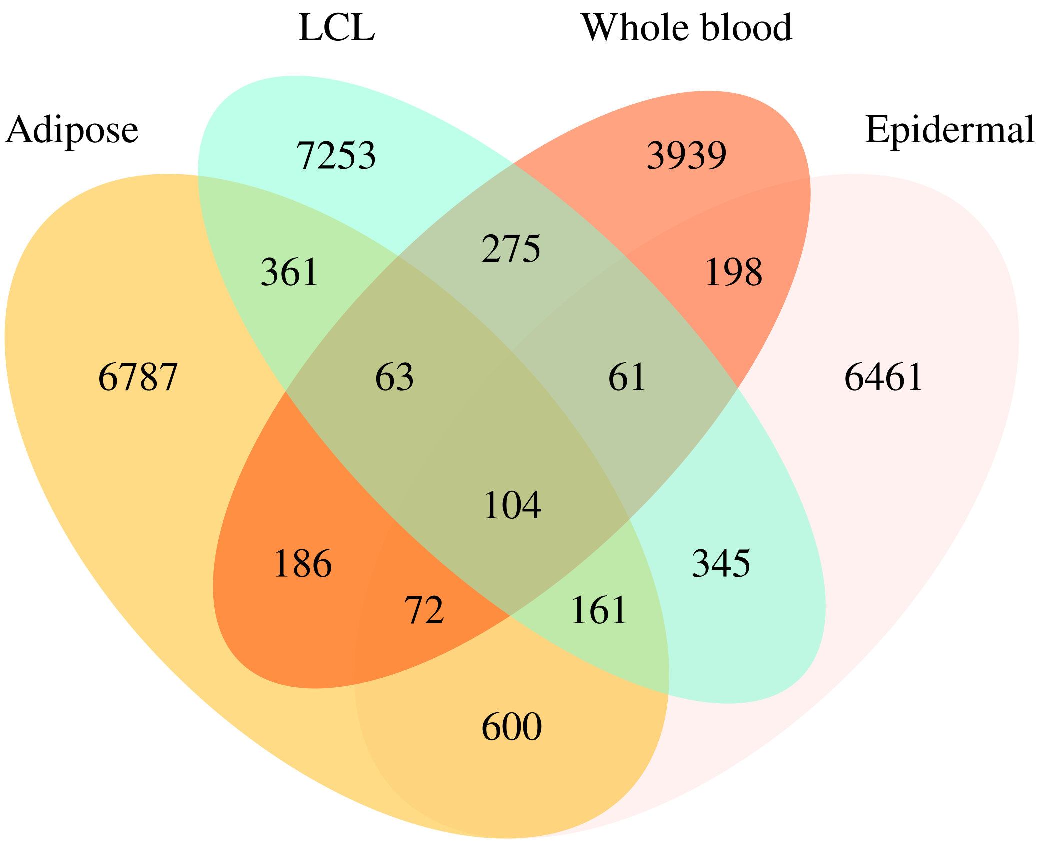

S11 Fig eQTL distribution across tissues. As established in [1], eQTLs have some tendency to act in more than one tissue.

1. Buil A, Viñuela A, Brown A, Davies M, Padioleau I, Bielser D, et al. Quantifying the degree of sharing of genetic and non-genetic causes of gene expression variability across four tissues. bioRxiv. 2016;doi:10.1101/053355.
