# Supplementary material for: Cross-tissue eQTL enrichment of associations in schizophrenia
Source: PLoS One. 2018 Sep 6;13(9):e0202812. doi: 10.1371/journal.pone.0202812 (PMC6126834; doi:10.1371/journal.pone.0202812)
Supplement: S1 Table — The test statistics refer to a general linear model of all brain and non-CNS variants in the ∼9 million variant template. (PDF) [file pone.0202812.s012.pdf]

S1 Table Schizophrenia association chi-squared general linear model coefficients for GTEx brain and GTEx/CommonMind consensus brain eQTLs compared to non-CNS eQTLs. The test statistics refer to a general linear model of all brain and non-CNS variants in the ∼9 million variant template. The π_1 are therefore overestimated and do not represent genome-wide values.

eQTL (n)		β	β (low 95%)	β (high 95%)	p	π_1	p_{MW}
Amygdala	GTEx (1046)	-0.059	-0.18	0.056	0.37	0.26	0.83
	GTEx&CM (591)	-0.048	-0.20	0.10	0.57	0.20	0.56
Cingulate	GTEx (1976)	-0.039	-0.12	0.046	0.42	0.38	0.68
	GTEx&CM (1055)	-0.025	-0.14	0.088	0.70	0.37	0.14
Caudate	GTEx (2861)	-0.056	-0.13	0.015	0.16	0.23	0.96
	GTEx&CM (1280)	-0.018	-0.12	0.085	0.76	0.30	0.12
Cerebellum	GTEx (4848)	0.028	-0.028	0.083	0.38	0.26	0.27
	GTEx&CM (1827)	0.063	-0.023	0.15	0.20	0.31	0.0017
Cortex	GTEx (3162)	-0.059	-0.13	0.009	0.13	0.33	0.92
	GTEx&CM (1620)	-0.024	-0.12	0.067	0.64	0.37	0.13
Frontal Cortex	GTEx (2454)	-0.026	-0.10	0.05	0.55	0.30	0.93
	GTEx&CM (1249)	0.031	-0.073	0.13	0.60	0.33	0.11
Hippocampus	GTEx (1547)	0.027	-0.068	0.12	0.61	0.15	0.93
	GTEx&CM (843)	0.083	-0.043	0.21	0.25	0.22	0.29
Hypothalamus	GTEx (1492)	-0.04	-0.14	0.057	0.47	0.24	0.95
	GTEx&CM (814)	0.036	-0.092	0.16	0.62	0.21	0.77
Nucleus accumbens	GTEx (2436)	-0.021	-0.098	0.056	0.63	0.32	0.62
	GTEx&CM (1155)	-0.026	-0.13	0.082	0.68	0.32	0.098
Putamen	GTEx (2022)	-0.045	-0.13	0.038	0.34	0.27	0.89
	GTEx&CM (993)	0.0031	-0.11	0.12	0.96	0.33	0.13
Substantia nigra	GTEx (825)	-0.082	-0.21	0.048	0.27	0.19	0.96
	GTEx&CM (478)	-0.057	-0.22	0.11	0.55	0.22	0.75
All	GTEx (20659)	0.013	-0.0069	0.034	0.19	0.26	0.96
	GTEx&CM (8992)	0.094	0.067	0.12	7.73E-12	0.30	0.00012
