# Supplementary material for: Cross-tissue eQTL enrichment of associations in schizophrenia
Source: PLoS One. 2018 Sep 6;13(9):e0202812. doi: 10.1371/journal.pone.0202812 (PMC6126834; doi:10.1371/journal.pone.0202812)
Supplement: S2 Table — (PDF) [file pone.0202812.s013.pdf]

S2 Table Schizophrenia association chi-squared differences between various eQTL types and matching control variants.

Adipose tissue eQTLs VS control

	Estimate	Std. Error	t value	Pr(>|t|)
Adipose eQTL	0.44	0.037	11.73	<2e-16 ***
control Adipose eQTL	-0.0079	0.021	-0.37	0.71

—

F-statistic: 68.85 on 2 and 2540800 DF, p-value: < 2.2e-16
Wilcoxon rank sum test: W = 22520000, p-value = 1.606e-11

Epidermal tissue eQTLs VS control

	Estimate	Std. Error	t value	Pr(>|t|)
Epidermal eQTL	0.41	0.04	10.27	<2e-16 ***
control Epidermal eQTL	0.011	0.021	0.52	0.61

—

F-statistic: 52.87 on 2 and 2540800 DF, p-value: < 2.2e-16
Wilcoxon rank sum test: W = 20528000, p-value = 4.364e-06

LCL eQTLs VS control

	Estimate	Std. Error	t value	Pr(>|t|)
LCL eQTL	0.53	0.035	15.15	< 2e-16 ***
control LCL eQTL	-0.065	0.023	-2.90	0.00374 **

—

F-statistic: 119.1 on 2 and 2540800 DF, p-value: < 2.2e-16
Wilcoxon rank sum test: W = 22572000, p-value = 1.039e-10

Whole blood eQTLs VS control

	Estimate	Std. Error	t value	Pr(>|t|)
Whole blood eQTL	0.49	0.046	10.49	< 2e-16 ***
control Whole blood eQTL	0.13	0.024	5.697	1.22e-08 ***

—

F-statistic: 71.18 on 2 and 2540800 DF, p-value: < 2.2e-16
Wilcoxon rank sum test: W = 11602000, p-value = 0.005409

proximal eQTLs VS distal eQTLs

	Estimate	Std. Error	t value	Pr(>|t|)
eQTL (prox)	0.49	0.029	17.24	<2e-16 ***
eQTL (dist)	0.46	0.026	17.32	<2e-16 ***

—

F-statistic: 297.9 on 2 and 2540800 DF, p-value: < 2.2e-16
Wilcoxon rank sum test: W = 23019000, p-value = 0.3818

promoter eQTLs VS non-promoter eQTLs

	Estimate	Std. Error	t value	Pr(>|t|)
eQTL (prom)	0.65	0.065	10.01	<2e-16 ***
eQTL (non-prom)	0.45	0.02	22.08	<2e-16 ***

—

F-statistic: 293.5 on 2 and 2540800 DF, p-value: < 2.2e-16
Wilcoxon rank sum test: W = 7765100, p-value = 0.03385

eQTLs VS control

	Estimate	Std. Error	t value	Pr(>|t|)
eQTL	0.47	0.02	24.06	<2e-16 ***
control eQTL	0.018	0.011	1.60	0.11

—

F-statistic: 290.5 on 2 and 2540800 DF, p-value: < 2.2e-16
Wilcoxon rank sum test: W = 288200000, p-value < 2.2e-16

Signif. codes: 0 '***' 0.001 '**' 0.01 '*' 0.05 '.' 0.1 ' ' 1
