# Supplementary material for: Cross-tissue eQTL enrichment of associations in schizophrenia
Source: PLoS One. 2018 Sep 6;13(9):e0202812. doi: 10.1371/journal.pone.0202812 (PMC6126834; doi:10.1371/journal.pone.0202812)
Supplement: S3 Table — (PDF) [file pone.0202812.s014.pdf]

S3 Table Total LD differences between various eQTL types and matching control variants.

Adipose tissue eQTLs VS control

	Estimate	Std. Error	t value	Pr(>|t|)
Adipose eQTL	5.09	1.82	2.80	0.00514 **
control Adipose eQTL	-24.48	1.04	-23.54	< 2e-16 ***

—
F-statistic: 281.1 on 2 and 2558408 DF, p-value: < 2.2e-16
Wilcoxon rank sum test: W = 26135000, p-value < 2.2e-16

Epidermal tissue eQTLs VS control

	Estimate	Std. Error	t value	Pr(>|t|)
Epidermal eQTL	6.06	1.92	3.15	0.00161 **
control Epidermal eQTL	-20.79	1.02	-20.37	< 2e-16 ***

—
F-statistic: 212.7 on 2 and 2558408 DF, p-value: < 2.2e-16
Wilcoxon rank sum test: W = 23586000, p-value < 2.2e-16

LCL eQTLs VS control

	Estimate	Std. Error	t value	Pr(>|t|)
LCL eQTL	11.08	1.71	6.46	1.03e-10 ***
control LCL eQTL	-27.44	1.10	-24.95	< 2e-16 ***

—
F-statistic: 332.4 on 2 and 2558408 DF, p-value: < 2.2e-16
Wilcoxon rank sum test: W = 27662000, p-value < 2.2e-16

Whole blood eQTLs VS control

	Estimate	Std. Error	t value	Pr(>|t|)
Whole blood eQTL	1.90	2.26	0.84	0.40
control Whole blood eQTL	-10.04	1.15	-8.74	<2e-16 ***

—
F-statistic: 38.6 on 2 and 2558408 DF, p-value: < 2.2e-16
Wilcoxon rank sum test: W = 12108000, p-value = 4.323e-09

proximal eQTLs VS distal eQTLs

	Estimate	Std. Error	t value	Pr(>|t|)
eQTL (prox)	-6.59	1.39	-4.73	2.25e-06 ***
eQTL (dist)	17.90	1.29	13.92	< 2e-16 ***

—
F-statistic: 108.2 on 2 and 2558408 DF, p-value: < 2.2e-16
Wilcoxon rank sum test: W = 19919000, p-value < 2.2e-16

promoter eQTLs VS non-promoter eQTLs

	Estimate	Std. Error	t value	Pr(>|t|)
eQTL (prom)	-0.59	3.16	-0.18	0.85
eQTL (non-prom)	7.44	1.00	7.46	8.3e-14 ***

—
F-statistic: 27.89 on 2 and 2558408 DF, p-value: 7.752e-13
Wilcoxon rank sum test: W = 7241200, p-value = 0.004889

eQTLs VS control

	Estimate	Std. Error	t value	Pr(>|t|)
eQTL	6.40	0.95	6.73	1.74e-11 ***
control eQTL	-20.43	0.56	-36.70	< 2e-16 ***

—
F-statistic: 698.3 on 2 and 2558408 DF, p-value: < 2.2e-16
Wilcoxon rank sum test: W = 329700000, p-value < 2.2e-16

Signif. codes: 0 '***' 0.001 '**' 0.01 '*' 0.05 '.' 0.1 ' ' 1
