# Supplementary material for: Cross-tissue eQTL enrichment of associations in schizophrenia
Source: PLoS One. 2018 Sep 6;13(9):e0202812. doi: 10.1371/journal.pone.0202812 (PMC6126834; doi:10.1371/journal.pone.0202812)
Supplement: S4 Table — All eQTLs designated by CommonMind or GTEx as brain eQTLs were excluded from these analyses. (PDF) [file pone.0202812.s015.pdf]

S4 Table Enrichment statistics and general linear model coefficients for squared schizophrenia association z-scores differences between adipose tissue, epidermal tissue, lymphoblastoid cell lines (LCL) and whole blood eQTLs, and matching control variants. All eQTLs designated by CommonMind or GTEx as brain eQTLs were excluded from these analyses.

annotation		$\bar{\beta}$	β (low 95%)	β (high 95%)	p	π_1	p_{MW}
Adipose	eQTL	0.12	0.043	0.20	0.0025	0.22	5.98E-07
	control	-0.08	-0.12	-0.039	0.00013	0.07	
Epidermal	eQTL	0.099	0.023	0.18	0.011	0.22	0.00027
	control	-0.076	-0.12	-0.031	0.0011	0.076	
LCL	eQTL	0.12	0.053	0.19	0.00058	0.12	1.53E-05
	control	-0.092	-0.13	-0.051	1.16E-05	0.082	
Whole blood	eQTL	0.15	0.05	0.25	0.0035	0.14	0.044
	control	-0.0015	-0.044	0.042	0.95	0.098	
All	prox	0.17	0.11	0.23	9.71E-09	0.16	0.69
	dist	0.089	0.036	0.14	0.00092	0.19	0.31
	eQTL	0.14	0.10	0.18	1.06E-11	0.17	3.24E-13

$\bar{\beta}$ is the mean effect size over the general linear model replicas with functional genetic affiliation covariates; p is the corresponding unadjusted p-value (see methods for more details); π_1 is the estimated proportion of non-null associations; p_{MW} is the unadjusted Mann-Whitney test p-value for differences in association chi-squared between eQTL and respective matched control variants; prox stands for proximal eQTLs, dist for distal eQTLs.
