# Supplementary material for: Cross-tissue eQTL enrichment of associations in schizophrenia
Source: PLoS One. 2018 Sep 6;13(9):e0202812. doi: 10.1371/journal.pone.0202812 (PMC6126834; doi:10.1371/journal.pone.0202812)
Supplement: S5 Table — (PDF) [file pone.0202812.s016.pdf]

S5 Table Schizophrenia association chi-squared general linear model coefficients for the four Roadmap functional affiliations.

annotation	β	β (95% low)	β (95% high)	p
Strong_Enhancer	0.039	0.022	0.055	3.58E-05
Weak_Enhancer	0.032	0.02	0.045	6.24E-06
Active_Promoter	0.21	0.18	0.24	9.04E-38
Weak_Promoter	0.13	0.11	0.16	2.31E-16
