# Supplementary material for: Cross-tissue eQTL enrichment of associations in schizophrenia
Source: PLoS One. 2018 Sep 6;13(9):e0202812. doi: 10.1371/journal.pone.0202812 (PMC6126834; doi:10.1371/journal.pone.0202812)
Supplement: S6 Table — (PDF) [file pone.0202812.s017.pdf]

S6 Table Schizophrenia association chi-squared general linear model coefficients for the four Roadmap functional affiliations restricted to eQTLs and control variants.

annotation	β	β (95% low)	β (95% high)	p
Strong_Enhancer	-0.024	-0.073	0.025	0.40
Weak_Enhancer	0.04	0.0011	0.08	0.071
Active_Promoter	0.099	0.052	0.15	0.00024
Weak_Promoter	0.082	0.022	0.14	0.016
