# Supplementary material for: Cross-tissue eQTL enrichment of associations in schizophrenia
Source: PLoS One. 2018 Sep 6;13(9):e0202812. doi: 10.1371/journal.pone.0202812 (PMC6126834; doi:10.1371/journal.pone.0202812)
Supplement: S7 Table — The test statistics refer to the respective interaction terms. The interaction with TotLD represents the enrichment ascribable to the eQTLs irrespective of their LD-tagging power. Enhancer and Promoter affiliations were assigned by Roadmap in the corresponding tissues. (PDF) [file pone.0202812.s018.pdf]

S7 Table Schizophrenia association chi-squared general linear model coefficients for tissue-specific eQTLs with different functional affiliations. The test statistics refer to the respective interaction terms. The interaction with TotLD represents the enrichment ascribable to the eQTLs irrespective of their LD-tagging power. Enhancer and Promoter affiliations were assigned by Roadmap in the corresponding tissues.

	annotation	β	β (95% low)	β (95% high)	p
Adipose eQTL	TotLD	0.054	0.021	0.086	0.0037
	Exon	0.049	0.017	0.082	0.0074
	Intron	0.032	-0.0022	0.067	0.10
	X5UTR	-0.018	-0.048	0.012	0.30
	X3UTR	-0.031	-0.066	0.0047	0.13
	Active_Promoter	-0.26	-0.47	-0.042	0.036
	Weak_Promoter	-0.014	-0.16	0.13	0.87
	Strong_Enhancer	0.05	-0.082	0.18	0.51
	Weak_Enhancer	-0.044	-0.21	0.12	0.65
Epidermal eQTL	TotLD	0.085	0.051	0.12	9.04E-06
	Exon	0.053	0.023	0.084	0.0023
	Intron	0.052	0.017	0.088	0.01
	X5UTR	0.025	-0.0096	0.059	0.21
	X3UTR	-0.017	-0.05	0.015	0.34
	Active_Promoter	-0.044	-0.22	0.13	0.67
	Weak_Promoter	0.17	-0.0069	0.35	0.092
	Strong_Enhancer	0.0031	-0.17	0.18	0.97
	Weak_Enhancer	-0.00035	-0.19	0.19	1.00
LCL eQTL	TotLD	0.07	0.035	0.10	0.00042
	Exon	0.017	-0.013	0.047	0.32
	Intron	0.035	0.0011	0.068	0.07
	X5UTR	-0.011	-0.042	0.02	0.53
	X3UTR	0.012	-0.018	0.043	0.48
	Active_Promoter	0.081	-0.062	0.22	0.32
	Weak_Promoter	-0.15	-0.35	0.052	0.20
	Strong_Enhancer	0.03	-0.11	0.17	0.72
	Weak_Enhancer	-0.028	-0.18	0.12	0.74
Whole blood eQTL	TotLD	0.087	0.04	0.13	0.0011
	Exon	0.031	-0.0077	0.07	0.16
	Intron	0.12	0.07	0.16	1.19E-05
	X5UTR	0.055	0.017	0.093	0.012
	X3UTR	-0.15	-0.18	-0.11	9.28E-12
	Active_Promoter	0.052	-0.37	0.47	0.83
	Weak_Promoter	0.12	-0.067	0.31	0.26
	Strong_Enhancer	0.13	-0.10	0.36	0.33
	Weak_Enhancer	-0.26	-0.53	0.015	0.097
