# Supplementary material for: Cross-tissue eQTL enrichment of associations in schizophrenia
Source: PLoS One. 2018 Sep 6;13(9):e0202812. doi: 10.1371/journal.pone.0202812 (PMC6126834; doi:10.1371/journal.pone.0202812)
Supplement: S8 Table — The test statistics refer to the respective interaction terms. The interaction with TotLD represents the enrichment ascribable to the eQTLs irrespective of their LD-tagging power. Enhancer and Promoter affiliations were assigned by Roadmap in the corresponding tissues. (PDF) [file pone.0202812.s019.pdf]

S8 Table Schizophrenia association chi-squared general linear model coefficients for all, proximal or distal eQTLs with different functional affiliations. The test statistics refer to the respective interaction terms. The interaction with TotLD represents the enrichment ascribable to the eQTLs irrespective of their LD-tagging power. Enhancer and Promoter affiliations were assigned by Roadmap in the corresponding tissues.

	annotation	β	β (95% low)	β (95% high)	p
Proximal eQTL	TotLD	0.21	0.17	0.24	6.81E-27
	Exon	0.047	0.023	0.072	0.0007
	Intron	0.14	0.11	0.18	2.71E-14
	X5UTR	0.031	0.0059	0.056	0.031
	X3UTR	-4.59E-05	-0.025	0.025	1.00
	Active_Promoter	-0.015	-0.12	0.095	0.81
	Weak_Promoter	0.088	-0.064	0.24	0.31
	Strong_Enhancer	-0.062	-0.20	0.071	0.41
	Weak_Enhancer	-2.62E-05	-0.12	0.12	1.00
Distal eQTL	TotLD	-0.023	-0.047	0.0011	0.094
	Exon	-0.009	-0.034	0.016	0.53
	Intron	-0.018	-0.042	0.007	0.21
	X5UTR	-0.041	-0.068	-0.014	0.0084
	X3UTR	-0.13	-0.15	-0.1	2.38E-16
	Active_Promoter	0.078	-0.10	0.26	0.45
	Weak_Promoter	0.034	-0.19	0.26	0.79
	Strong_Enhancer	0.094	-0.046	0.23	0.24
	Weak_Enhancer	-0.14	-0.27	-0.0082	0.062
All eQTL	TotLD	0.064	0.043	0.084	9.26E-08
	Exon	0.028	0.008	0.047	0.014
	Intron	0.046	0.025	0.067	0.00014
	X5UTR	0.0037	-0.017	0.024	0.76
	X3UTR	-0.059	-0.079	-0.038	3.55E-07
	Active_Promoter	0.0047	-0.096	0.11	0.94
	Weak_Promoter	0.083	-0.052	0.22	0.28
	Strong_Enhancer	0.032	-0.075	0.14	0.60
	Weak_Enhancer	-0.055	-0.15	0.04	0.31
