# Supplementary material for: Cross-tissue eQTL enrichment of associations in schizophrenia
Source: PLoS One. 2018 Sep 6;13(9):e0202812. doi: 10.1371/journal.pone.0202812 (PMC6126834; doi:10.1371/journal.pone.0202812)
Supplement: S9 Table — The test statistics refer to the respective interaction terms. The interaction with TotLD represents the enrichment ascribable to the eQTLs irrespective of their LD-tagging power. Enhancer and Promoter affiliations were assigned by Roadmap in the corresponding tissues. (PDF) [file pone.0202812.s020.pdf]

S9 Table Schizophrenia association chi-squared general linear model coefficients for tissue-specific eQTLs with different functional affiliations upon exclusion of CommonMind and GTEx brain eQTLs. The test statistics refer to the respective interaction terms. The interaction with TotLD represents the enrichment ascribable to the eQTLs irrespective of their LD-tagging power. Enhancer and Promoter affiliations were assigned by Roadmap in the corresponding tissues.

	annotation	β	β (95% low)	β (95% high)	p
Adipose eQTL	TotLD	0.0073	-0.028	0.042	0.72
	Exon	0.025	-0.012	0.062	0.23
	Intron	0.008	-0.029	0.045	0.71
	X5UTR	-0.017	-0.055	0.02	0.42
	X3UTR	-0.06	-0.10	-0.02	0.0085
	Active_Promoter	-0.10	-0.36	0.16	0.50
	Weak_Promoter	-0.044	-0.21	0.13	0.65
	Strong_Enhancer	-0.017	-0.16	0.13	0.83
	Weak_Enhancer	-0.15	-0.33	0.026	0.13
Epidermal eQTL	TotLD	0.08	0.045	0.12	6.74E-05
	Exon	0.049	0.018	0.081	0.0062
	Intron	0.046	0.009	0.083	0.029
	X5UTR	0.028	-0.0065	0.063	0.15
	X3UTR	-0.029	-0.062	0.0037	0.12
	Active_Promoter	0.045	-0.15	0.24	0.68
	Weak_Promoter	0.077	-0.11	0.27	0.48
	Strong_Enhancer	0.033	-0.14	0.21	0.74
	Weak_Enhancer	-0.019	-0.21	0.17	0.86
LCL eQTL	TotLD	0.019	-0.017	0.055	0.35
	Exon	-0.019	-0.051	0.013	0.30
	Intron	0.0027	-0.032	0.037	0.89
	X5UTR	-0.038	-0.071	-0.0057	0.04
	X3UTR	-0.023	-0.057	0.01	0.22
	Active_Promoter	0.056	-0.10	0.21	0.53
	Weak_Promoter	-0.17	-0.38	0.049	0.17
	Strong_Enhancer	0.024	-0.13	0.17	0.78
	Weak_Enhancer	0.004	-0.15	0.16	0.96
Whole blood eQTL	TotLD	0.072	0.018	0.12	0.018
	Exon	-0.014	-0.058	0.03	0.59
	Intron	0.096	0.044	0.15	0.0012
	X5UTR	0.087	0.034	0.14	0.0039
	X3UTR	-0.14	-0.18	-0.093	1.98E-08
	Active_Promoter	-0.0093	-0.56	0.54	0.98
	Weak_Promoter	0.16	-0.07	0.39	0.22
	Strong_Enhancer	0.029	-0.23	0.29	0.85
	Weak_Enhancer	-0.11	-0.42	0.21	0.55
