# Supplementary material for: Cross-tissue eQTL enrichment of associations in schizophrenia
Source: PLoS One. 2018 Sep 6;13(9):e0202812. doi: 10.1371/journal.pone.0202812 (PMC6126834; doi:10.1371/journal.pone.0202812)
Supplement: S10 Table — The test statistics refer to the respective interaction terms. The interaction with TotLD represents the enrichment ascribable to the eQTLs irrespective of their LD-tagging power. Enhancer and Promoter affiliations were assigned by Roadmap in the corresponding tissues. (PDF) [file pone.0202812.s021.pdf]

S10 Table Schizophrenia association chi-squared general linear model coefficients for all, proximal or distal eQTLs with different functional affiliations upon exclusion of CommonMind and GTEx brain eQTLs. The test statistics refer to the respective interaction terms. The interaction with TotLD represents the enrichment ascribable to the eQTLs irrespective of their LD-tagging power. Enhancer and Promoter affiliations were assigned by Roadmap in the corresponding tissues.

	annotation	β	β (95% low)	β (95% high)	p
	TotLD	0.19	0.15	0.22	7.32E-22
	Exon	0.046	0.021	0.072	0.0013
	Intron	0.14	0.10	0.17	1.57E-12
	X5UTR	0.035	0.0095	0.061	0.016
Proximal eQTL	X3UTR	-0.012	-0.037	0.014	0.42
	Active_Promoter	0.0049	-0.11	0.12	0.94
	Weak_Promoter	0.16	0.0048	0.32	0.071
	Strong_Enhancer	-0.084	-0.22	0.052	0.28
	Weak_Enhancer	-0.0014	-0.12	0.12	0.98
	TotLD	-0.025	-0.049	-0.00063	0.072
	Exon	-0.012	-0.038	0.014	0.41
	Intron	-0.019	-0.044	0.0063	0.19
	X5UTR	-0.037	-0.065	-0.0099	0.017
Distal eQTL	X3UTR	-0.13	-0.16	-0.10	1.92E-16
	Active_Promoter	0.13	-0.059	0.32	0.23
	Weak_Promoter	0.0021	-0.23	0.23	0.99
	Strong_Enhancer	0.089	-0.052	0.23	0.27
	Weak_Enhancer	-0.13	-0.26	0.00083	0.082
	TotLD	0.064	0.043	0.084	9.26E-08
	Exon	0.028	0.008	0.047	0.014
	Intron	0.046	0.025	0.067	0.00014
	X5UTR	0.0037	-0.017	0.024	0.76
All eQTL	X3UTR	-0.059	-0.079	-0.038	3.55E-07
	Active_Promoter	0.0047	-0.096	0.11	0.94
	Weak_Promoter	0.083	-0.052	0.22	0.28
	Strong_Enhancer	0.032	-0.075	0.14	0.60
	Weak_Enhancer	-0.055	-0.15	0.04	0.31
