# Supplementary material for: Cross-tissue eQTL enrichment of associations in schizophrenia
Source: PLoS One. 2018 Sep 6;13(9):e0202812. doi: 10.1371/journal.pone.0202812 (PMC6126834; doi:10.1371/journal.pone.0202812)
Supplement: S11 Table — (PDF) [file pone.0202812.s022.pdf]

S11 Table Enrichment statistics and general linear model coefficients for squared BMI association z-scores differences between adipose tissue, epidermal tissue, lymphoblastoid cell lines (LCL) and whole blood eQTLs, and matching control variants.

annotation		$\bar{\beta}$	β (low 95%)	β (high 95%)	p	π_1	p_{MW}
Adipose	eQTL	0.13	0.059	0.20	0.00032	0.22	2.38E-08
	control	-0.043	-0.084	-0.003	0.035	0.072	
Epidermal	eQTL	0.12	0.049	0.18	0.00067	0.15	7.96E-07
	control	-0.041	-0.084	0.0018	0.06	0.048	
LCL	eQTL	0.12	0.05	0.18	0.00061	0.13	3.34E-09
	control	-0.093	-0.14	-0.051	1.45E-05	0.035	
Whole blood	eQTL	0.15	-0.0049	0.30	0.058	0.093	1.72E-05
	control	-0.018	-0.062	0.026	0.43	0.027	
All	prox	0.095	0.044	0.15	0.00025	0.13	0.97
	dist	0.18	0.13	0.23	2.98E-13	0.17	0.034
	eQTL	0.17	0.13	0.20	1.20E-17	0.15	7.74E-24

$\bar{\beta}$ is the mean effect size over the general linear model replicas with functional genetic affiliation covariates; p is the corresponding unadjusted p-value (see methods for more details); π_1 is the estimated proportion of non-null associations; p_{MW} is the unadjusted Mann-Whitney test p-value for differences in association chi-squared between eQTL and respective matched control variants; prox stands for proximal eQTLs, dist for distal eQTLs.
