# Supplementary material for: Cross-tissue eQTL enrichment of associations in schizophrenia
Source: PLoS One. 2018 Sep 6;13(9):e0202812. doi: 10.1371/journal.pone.0202812 (PMC6126834; doi:10.1371/journal.pone.0202812)
Supplement: S12 Table — Enhancer and Promoter affiliations were assigned by Roadmap in the corresponding tissues. (PDF) [file pone.0202812.s023.pdf]

S12 Table BMI association chi-squared general linear model coefficients for all eQTL types with the four Roadmap functional affiliations. Enhancer and Promoter affiliations were assigned by Roadmap in the corresponding tissues.

	annotation	β	β (95% low)	β (95% high)	p
	Strong_Enhancer	0.0061	-0.093	0.11	0.91
	Weak_Enhancer	-0.072	-0.16	0.013	0.14
	Active_Promoter	4.38E-05	-0.11	0.11	1.00
	Weak_Promoter	-0.069	-0.20	0.064	0.36
Adipose eQTL	Active_Promoter	-0.019	-0.52	0.48	0.95
	Weak_Promoter	-0.072	-0.38	0.24	0.68
	Strong_Enhancer	0.024	-0.22	0.27	0.87
	Weak_Enhancer	-0.11	-0.44	0.22	0.57
Epidermal eQTL	Active_Promoter	-0.17	-0.59	0.25	0.49
	Weak_Promoter	0.12	-0.26	0.51	0.58
	Strong_Enhancer	-0.0027	-0.32	0.31	0.99
	Weak_Enhancer	0.12	-0.23	0.47	0.56
LCL eQTL	Active_Promoter	-0.0088	-0.32	0.31	0.96
	Weak_Promoter	-0.25	-0.67	0.17	0.30
	Strong_Enhancer	-0.17	-0.45	0.11	0.29
	Weak_Enhancer	-0.023	-0.32	0.28	0.89
Whole blood eQTL	Active_Promoter	-0.12	-1.13	0.90	0.84
	Weak_Promoter	-0.026	-0.42	0.37	0.91
	Strong_Enhancer	-0.085	-0.54	0.37	0.74
	Weak_Enhancer	0.029	-0.50	0.56	0.92
Proximal eQTL	Active_Promoter	-0.067	-0.31	0.18	0.63
	Weak_Promoter	0.17	-0.15	0.50	0.35
	Strong_Enhancer	-0.015	-0.28	0.25	0.92
	Weak_Enhancer	0.0041	-0.25	0.26	0.98
Distal eQTL	Active_Promoter	0.02	-0.40	0.44	0.93
	Weak_Promoter	-0.44	-0.93	0.058	0.12
	Strong_Enhancer	0.032	-0.23	0.30	0.83
	Weak_Enhancer	-0.27	-0.53	-0.0027	0.077
All eQTL	Active_Promoter	-0.10	-0.33	0.13	0.43
	Weak_Promoter	-0.038	-0.33	0.26	0.82
	Strong_Enhancer	-0.038	-0.25	0.17	0.75
	Weak_Enhancer	-0.14	-0.34	0.056	0.21
