# Supplementary material for: Cross-tissue eQTL enrichment of associations in schizophrenia
Source: PLoS One. 2018 Sep 6;13(9):e0202812. doi: 10.1371/journal.pone.0202812 (PMC6126834; doi:10.1371/journal.pone.0202812)
Supplement: S13 Table — (PDF) [file pone.0202812.s024.pdf]

S13 Table Enrichment statistics and general linear model coefficients for squared Height association z-scores differences between adipose tissue, epidermal tissue, lymphoblastoid cell lines (LCL) and whole blood eQTLs, and matching control variants.

annotation		$\bar{\beta}$	β (low 95%)	β (high 95%)	p	π_1	p_{MW}
Adipose	eQTL	0.19	0.055	0.32	0.0057	0.20	4.77E-08
	control	-0.093	-0.15	-0.037	0.0011	0.17	
Epidermal	eQTL	0.16	0.056	0.26	0.0026	0.24	5.88E-08
	control	-0.058	-0.11	-0.0024	0.041	0.20	
LCL	eQTL	0.30	0.18	0.43	3.19E-06	0.24	3.06E-16
	control	-0.18	-0.24	-0.12	1.51E-09	0.14	
Whole blood	eQTL	0.18	0.04	0.33	0.012	0.22	0.00032
	control	-0.0077	-0.071	0.055	0.81	0.16	
All	prox	0.24	0.17	0.31	1.04E-11	0.25	0.023
	dist	0.26	0.17	0.34	1.16E-08	0.21	0.98
	eQTL	0.28	0.22	0.34	4.26E-21	0.22	6.86E-29

$\bar{\beta}$ is the mean effect size over the general linear model replicas with functional genetic affiliation covariates; p is the corresponding unadjusted p-value (see methods for more details); π_1 is the estimated proportion of non-null associations; p_{MW} is the unadjusted Mann-Whitney test p-value for differences in association chi-squared between eQTL and respective matched control variants; prox stands for proximal eQTLs, dist for distal eQTLs.
