# Supplementary material for: Cross-tissue eQTL enrichment of associations in schizophrenia
Source: PLoS One. 2018 Sep 6;13(9):e0202812. doi: 10.1371/journal.pone.0202812 (PMC6126834; doi:10.1371/journal.pone.0202812)
Supplement: S14 Table — Enhancer and Promoter affiliations were assigned by Roadmap in the corresponding tissues. (PDF) [file pone.0202812.s025.pdf]

S14 Table Height association chi-squared general linear model coefficients for all eQTL types with the four Roadmap functional affiliations. Enhancer and Promoter affiliations were assigned by Roadmap in the corresponding tissues.

	annotation	β	β (95% low)	β (95% high)	p
	Strong_Enhancer	0.16	0.031	0.28	0.029
	Weak_Enhancer	0.11	0.0051	0.22	0.066
	Active_Promoter	0.11	-0.023	0.24	0.15
	Weak_Promoter	0.1	-0.066	0.27	0.29
Adipose eQTL	Active_Promoter	-0.18	-0.81	0.45	0.62
	Weak_Promoter	-0.31	-0.69	0.083	0.17
	Strong_Enhancer	-0.23	-0.54	0.084	0.20
	Weak_Enhancer	0.19	-0.22	0.60	0.42
Epidermal eQTL	Active_Promoter	0.028	-0.50	0.56	0.93
	Weak_Promoter	0.21	-0.27	0.70	0.44
	Strong_Enhancer	0.067	-0.33	0.46	0.77
	Weak_Enhancer	0.11	-0.33	0.56	0.66
LCL eQTL	Active_Promoter	-0.33	-0.72	0.068	0.15
	Weak_Promoter	0.60	0.078	1.13	0.044
	Strong_Enhancer	-0.36	-0.71	-0.01	0.071
	Weak_Enhancer	0.042	-0.34	0.42	0.85
Whole blood eQTL	Active_Promoter	-0.60	-1.88	0.67	0.41
	Weak_Promoter	0.25	-0.24	0.75	0.37
	Strong_Enhancer	-0.45	-1.02	0.12	0.17
	Weak_Enhancer	-0.29	-0.95	0.37	0.44
Proximal eQTL	Active_Promoter	-0.057	-0.37	0.25	0.75
	Weak_Promoter	0.64	0.23	1.05	0.0064
	Strong_Enhancer	-0.075	-0.40	0.25	0.69
	Weak_Enhancer	-0.22	-0.54	0.092	0.21
Distal eQTL	Active_Promoter	-0.39	-0.92	0.13	0.19
	Weak_Promoter	-0.043	-0.66	0.58	0.90
	Strong_Enhancer	-0.069	-0.40	0.27	0.72
	Weak_Enhancer	0.36	0.022	0.69	0.061
All eQTL	Active_Promoter	-0.19	-0.48	0.098	0.25
	Weak_Promoter	0.43	0.059	0.80	0.042
	Strong_Enhancer	-0.065	-0.33	0.20	0.67
	Weak_Enhancer	0.067	-0.18	0.32	0.64
