# Supplementary material for: Cross-tissue eQTL enrichment of associations in schizophrenia
Source: PLoS One. 2018 Sep 6;13(9):e0202812. doi: 10.1371/journal.pone.0202812 (PMC6126834; doi:10.1371/journal.pone.0202812)
Supplement: S15 Table — (PDF) [file pone.0202812.s026.pdf]

S15 Table Enrichment statistics and general linear model coefficients for squared RA association z-scores differences between adipose tissue, epidermal tissue, lymphoblastoid cell lines (LCL) and whole blood eQTLs, and matching control variants.

annotation		$\bar{\beta}$	β (low 95%)	β (high 95%)	p	π_1	p_{MW}
Adipose	eQTL	0.34	-0.0013	0.67	0.051	0.12	0.00011
	control	-0.087	-0.20	0.029	0.14	0.012	
Epidermal	eQTL	0.27	-0.087	0.64	0.14	0.12	4.58E-05
	control	-0.14	-0.25	-0.023	0.018	0.058	
LCL	eQTL	0.52	0.28	0.75	1.62E-05	0.099	3.11E-09
	control	-0.18	-0.30	-0.062	0.003	0.0094	
Whole blood	eQTL	-0.11	-0.38	0.15	0.40	0.14	0.045
	control	-0.047	-0.17	0.079	0.46	0.096	
All	prox	0.47	0.12	0.81	0.0085	0.096	0.90
	dist	0.18	-0.086	0.44	0.19	0.13	0.10
	eQTL	0.37	0.26	0.47	4.78E-11	0.12	1.41E-14

$\bar{\beta}$ is the mean effect size over the general linear model replicas with functional genetic affiliation covariates; p is the corresponding unadjusted p-value (see methods for more details); π_1 is the estimated proportion of non-null associations; p_{MW} is the unadjusted Mann-Whitney test p-value for differences in association chi-squared between eQTL and respective matched control variants; prox stands for proximal eQTLs, dist for distal eQTLs.
