# Supplementary material for: Cross-tissue eQTL enrichment of associations in schizophrenia
Source: PLoS One. 2018 Sep 6;13(9):e0202812. doi: 10.1371/journal.pone.0202812 (PMC6126834; doi:10.1371/journal.pone.0202812)
Supplement: S16 Table — Enhancer and Promoter affiliations were assigned by Roadmap in the corresponding tissues. (PDF) [file pone.0202812.s027.pdf]

S16 Table Rheumatoid arthritis association chi-squared general linear model coefficients for all eQTL types with the four Roadmap functional affiliations. Enhancer and Promoter affiliations were assigned by Roadmap in the corresponding tissues.

	annotation	β	β (95% low)	β (95% high)	p
	Strong_Enhancer	1.02	0.85	1.18	2.22E-27
	Weak_Enhancer	0.26	0.13	0.39	0.00058
	Active_Promoter	0.20	0.037	0.35	0.03
	Weak_Promoter	0.0018	-0.20	0.20	0.99
Adipose eQTL	Active_Promoter	0.71	-0.026	1.44	0.091
	Weak_Promoter	0.034	-0.46	0.53	0.90
	Strong_Enhancer	0.13	-0.31	0.58	0.61
	Weak_Enhancer	-0.0087	-0.57	0.56	0.98
Epidermal eQTL	Active_Promoter	0.07	-0.55	0.69	0.84
	Weak_Promoter	-0.70	-1.32	-0.085	0.046
	Strong_Enhancer	-0.84	-1.42	-0.26	0.011
	Weak_Enhancer	-0.68	-1.31	-0.051	0.058
LCL eQTL	Active_Promoter	0.019	-0.47	0.51	0.95
	Weak_Promoter	0.47	-0.21	1.14	0.23
	Strong_Enhancer	1.34	0.85	1.83	1.67E-06
	Weak_Enhancer	-0.42	-0.93	0.08	0.14
Whole blood eQTL	Active_Promoter	0.17	-1.28	1.62	0.84
	Weak_Promoter	0.013	-0.63	0.65	0.97
	Strong_Enhancer	-0.12	-0.91	0.66	0.78
	Weak_Enhancer	-0.014	-0.93	0.90	0.98
Proximal eQTL	Active_Promoter	0.40	0.023	0.78	0.063
	Weak_Promoter	0.20	-0.32	0.72	0.49
	Strong_Enhancer	0.98	0.53	1.43	0.00012
	Weak_Enhancer	-0.19	-0.60	0.22	0.41
Distal eQTL	Active_Promoter	-0.15	-0.79	0.48	0.68
	Weak_Promoter	-0.43	-1.22	0.36	0.34
	Strong_Enhancer	1.27	0.79	1.74	3.14E-06
	Weak_Enhancer	0.73	0.28	1.18	0.0041
All eQTL	Active_Promoter	0.25	-0.099	0.60	0.21
	Weak_Promoter	0.017	-0.45	0.48	0.95
	Strong_Enhancer	1.40	1.04	1.76	1.37E-11
	Weak_Enhancer	0.28	-0.046	0.60	0.13
