# Supplementary material for: Cross-tissue eQTL enrichment of associations in schizophrenia
Source: PLoS One. 2018 Sep 6;13(9):e0202812. doi: 10.1371/journal.pone.0202812 (PMC6126834; doi:10.1371/journal.pone.0202812)
Supplement: S17 Table — (PDF) [file pone.0202812.s028.pdf]

S17 Table Enrichment statistics and general linear model coefficients for squared SBP association z-scores differences between adipose tissue, epidermal tissue, lymphoblastoid cell lines (LCL) and whole blood eQTLs, and matching control variants.

annotation		$\bar{\beta}$	β (low 95%)	β (high 95%)	p	π_1	p_{MW}
Adipose	eQTL	0.081	-0.0023	0.16	0.057	0.11	0.00036
	control	-0.018	-0.054	0.019	0.34	0.069	
Epidermal	eQTL	0.06	-0.0051	0.13	0.071	0.15	0.016
	control	-0.021	-0.057	0.016	0.26	0.098	
LCL	eQTL	0.038	-0.019	0.094	0.19	0.06	0.049
	control	-0.031	-0.069	0.0072	0.11	0.047	
Whole blood	eQTL	0.097	0.022	0.17	0.011	0.077	0.089
	control	-0.025	-0.065	0.015	0.22	0.089	
All	prox	0.072	0.026	0.12	0.0024	0.11	0.83
	dist	0.082	0.032	0.13	0.0013	0.10	0.17
	eQTL	0.085	0.05	0.12	1.53E-06	0.1	9.52E-06

$\bar{\beta}$ is the mean effect size over the general linear model replicas with functional genetic affiliation covariates; p is the corresponding unadjusted p-value (see methods for more details); π_1 is the estimated proportion of non-null associations; p_{MW} is the unadjusted Mann-Whitney test p-value for differences in association chi-squared between eQTL and respective matched control variants; prox stands for proximal eQTLs, dist for distal eQTLs.
