# Supplementary material for: Cross-tissue eQTL enrichment of associations in schizophrenia
Source: PLoS One. 2018 Sep 6;13(9):e0202812. doi: 10.1371/journal.pone.0202812 (PMC6126834; doi:10.1371/journal.pone.0202812)
Supplement: S18 Table — Enhancer and Promoter affiliations were assigned by Roadmap in the corresponding tissues. (PDF) [file pone.0202812.s029.pdf]

S18 Table Systolic blood pressure association chi-squared general linear model coefficients for all eQTL types with the four Roadmap functional affiliations. Enhancer and Promoter affiliations were assigned by Roadmap in the corresponding tissues.

	annotation	β	β (95% low)	β (95% high)	p
	Strong_Enhancer	0.059	-0.0058	0.12	0.11
	Weak_Enhancer	0.019	-0.036	0.075	0.54
	Active_Promoter	-0.0011	-0.073	0.07	0.98
	Weak_Promoter	-0.071	-0.16	0.017	0.16
Adipose eQTL	Active_Promoter	-0.21	-0.55	0.13	0.29
	Weak_Promoter	0.00054	-0.21	0.21	1.00
	Strong_Enhancer	-0.035	-0.20	0.13	0.70
	Weak_Enhancer	0.27	0.059	0.48	0.025
Epidermal eQTL	Active_Promoter	-0.11	-0.40	0.18	0.51
	Weak_Promoter	-0.18	-0.43	0.079	0.23
	Strong_Enhancer	0.16	-0.043	0.37	0.17
	Weak_Enhancer	0.081	-0.15	0.31	0.53
LCL eQTL	Active_Promoter	0.17	-0.045	0.38	0.17
	Weak_Promoter	-0.14	-0.42	0.14	0.39
	Strong_Enhancer	0.067	-0.11	0.25	0.52
	Weak_Enhancer	0.029	-0.17	0.23	0.80
Whole blood eQTL	Active_Promoter	-0.38	-1.08	0.33	0.35
	Weak_Promoter	-0.19	-0.45	0.075	0.21
	Strong_Enhancer	0.16	-0.13	0.46	0.34
	Weak_Enhancer	0.032	-0.31	0.37	0.87
Proximal eQTL	Active_Promoter	0.016	-0.15	0.18	0.87
	Weak_Promoter	-0.097	-0.31	0.12	0.43
	Strong_Enhancer	-0.011	-0.18	0.16	0.91
	Weak_Enhancer	-0.077	-0.24	0.089	0.42
Distal eQTL	Active_Promoter	-0.15	-0.43	0.13	0.36
	Weak_Promoter	0.074	-0.25	0.40	0.69
	Strong_Enhancer	-0.028	-0.20	0.15	0.78
	Weak_Enhancer	0.13	-0.045	0.30	0.19
All eQTL	Active_Promoter	-0.011	-0.17	0.14	0.90
	Weak_Promoter	-0.079	-0.27	0.12	0.48
	Strong_Enhancer	-0.021	-0.16	0.12	0.79
	Weak_Enhancer	0.027	-0.10	0.16	0.72
