# Supplementary material for: Cross-tissue eQTL enrichment of associations in schizophrenia
Source: PLoS One. 2018 Sep 6;13(9):e0202812. doi: 10.1371/journal.pone.0202812 (PMC6126834; doi:10.1371/journal.pone.0202812)
Supplement: S19 Table — (PDF) [file pone.0202812.s030.pdf]

S19 Table Enrichment statistics and general linear model coefficients for squared T2D association z-scores differences between adipose tissue, epidermal tissue, lymphoblastoid cell lines (LCL) and whole blood eQTLs, and matching control variants.

annotation		$\bar{\beta}$	β (low 95%)	β (high 95%)	p	π_1	p_{MW}
Adipose	eQTL	0.13	0.054	0.20	0.00059	0.13	0.00049
	control	-0.043	-0.082	-0.005	0.027	0.017	
Epidermal	eQTL	-0.011	-0.089	0.066	0.78	0.068	0.081
	control	-0.0019	-0.067	0.063	0.95	0.048	
LCL	eQTL	0.039	-0.055	0.13	0.42	0.041	0.13
	control	-0.0043	-0.044	0.036	0.83	0.035	
Whole blood	eQTL	-0.0032	-0.081	0.074	0.94	0	0.75
	control	-0.0024	-0.05	0.045	0.92	0.098	
All	prox	0.013	-0.048	0.075	0.67	0.038	0.85
	dist	0.072	0.026	0.12	0.0023	0.082	0.15
	eQTL	0.055	0.0085	0.10	0.02	0.066	0.003

$\bar{\beta}$ is the mean effect size over the general linear model replicas with functional genetic affiliation covariates; p is the corresponding unadjusted p-value (see methods for more details); π_1 is the estimated proportion of non-null associations; p_{MW} is the unadjusted Mann-Whitney test p-value for differences in association chi-squared between eQTL and respective matched control variants; prox stands for proximal eQTLs, dist for distal eQTLs.
