# Supplementary material for: Cross-tissue eQTL enrichment of associations in schizophrenia
Source: PLoS One. 2018 Sep 6;13(9):e0202812. doi: 10.1371/journal.pone.0202812 (PMC6126834; doi:10.1371/journal.pone.0202812)
Supplement: S20 Table — Enhancer and Promoter affiliations were assigned by Roadmap in the corresponding tissues. (PDF) [file pone.0202812.s031.pdf]

S20 Table Type-II diabetes association chi-squared general linear model coefficients for all eQTL types with the four Roadmap functional affiliations. Enhancer and Promoter affiliations were assigned by Roadmap in the corresponding tissues.

	annotation	β	β (95% low)	β (95% high)	p
	Strong_Enhancer	-0.024	-0.089	0.041	0.51
	Weak_Enhancer	-0.013	-0.068	0.043	0.69
	Active_Promoter	0.0025	-0.069	0.074	0.95
	Weak_Promoter	-0.00032	-0.088	0.087	0.99
Adipose eQTL	Active_Promoter	-0.38	-0.72	-0.041	0.049
	Weak_Promoter	-0.12	-0.32	0.091	0.32
	Strong_Enhancer	0.12	-0.043	0.28	0.20
	Weak_Enhancer	0.25	0.038	0.47	0.039
Epidermal eQTL	Active_Promoter	-0.19	-0.48	0.10	0.25
	Weak_Promoter	-0.20	-0.46	0.054	0.17
	Strong_Enhancer	-0.035	-0.24	0.17	0.77
	Weak_Enhancer	-0.045	-0.28	0.18	0.73
LCL eQTL	Active_Promoter	-0.013	-0.22	0.20	0.92
	Weak_Promoter	0.062	-0.22	0.34	0.70
	Strong_Enhancer	-0.20	-0.38	-0.022	0.05
	Weak_Enhancer	0.077	-0.12	0.27	0.49
Whole blood eQTL	Active_Promoter	-0.39	-1.07	0.30	0.32
	Weak_Promoter	0.13	-0.13	0.40	0.37
	Strong_Enhancer	-0.16	-0.46	0.14	0.35
	Weak_Enhancer	0.22	-0.12	0.57	0.25
Proximal eQTL	Active_Promoter	-0.0085	-0.18	0.16	0.93
	Weak_Promoter	-0.15	-0.36	0.069	0.23
	Strong_Enhancer	-0.049	-0.22	0.12	0.62
	Weak_Enhancer	0.019	-0.15	0.18	0.84
Distal eQTL	Active_Promoter	-0.17	-0.45	0.11	0.28
	Weak_Promoter	-0.12	-0.45	0.20	0.51
	Strong_Enhancer	-0.17	-0.35	0.00087	0.081
	Weak_Enhancer	0.11	-0.068	0.28	0.29
All eQTL	Active_Promoter	-0.075	-0.23	0.08	0.40
	Weak_Promoter	-0.17	-0.37	0.022	0.12
	Strong_Enhancer	-0.13	-0.27	0.011	0.11
	Weak_Enhancer	0.074	-0.056	0.20	0.32
