# Supplementary material for: Cross-tissue eQTL enrichment of associations in schizophrenia
Source: PLoS One. 2018 Sep 6;13(9):e0202812. doi: 10.1371/journal.pone.0202812 (PMC6126834; doi:10.1371/journal.pone.0202812)
Supplement: S21 Table — The eQTLs and control variants from [25] were projected onto templates of ∼2.5 million variants (∼9 million variants for analyses involving brain eQTLs) with known pairwise LD. (PDF) [file pone.0202812.s032.pdf]

S21 Table eQTLs and matched control variants census in the data set used. The eQTLs and control variants from [1] were projected onto templates of ∼2.5 million variants (∼9 million variants for analyses involving brain eQTLs) with known pairwise LD.

Tissue	eQTL<br>2m (9m/Brain*)	Control<br>2m (9m)
Adipose	4193 (8334/564)	11389 (29385)
Epidermal	4130 (8002/579)	11831 (30072)
LCL	4441 (8623/480)	10172 (26604)
Whole blood	2406 (4898/336)	9341 (20385)
**All**	13624 (26866/1399)	40194 (101990)
**Brain****	(8992)	-

* GTEx [2] eQTLs
** GTEx [2] and CommonMind [3] consensus set

1. Buil A, Viñuela A, Brown A, Davies M, Padioleau I, Bielser D, et al. Quantifying the degree of sharing of genetic and non-genetic causes of gene expression variability across four tissues. bioRxiv. 2016;doi:10.1101/053355.

2. GTEx Consortium. The Genotype-Tissue Expression (GTEx) pilot analysis: Multitissue gene regulation in humans. Science. 2015;348(6235):648–660. doi:10.1126/science.1262110.

3. Fromer M, Roussos P, Sieberts SK, Johnson JS, Kavanagh DH, Perumal TM, et al. Gene expression elucidates functional impact of polygenic risk for schizophrenia. Nature Neuroscience. 2016;.
