# Supplementary material for: Cross-tissue eQTL enrichment of associations in schizophrenia
Source: PLoS One. 2018 Sep 6;13(9):e0202812. doi: 10.1371/journal.pone.0202812 (PMC6126834; doi:10.1371/journal.pone.0202812)
Supplement: S22 Table — The numbers refer to the ∼9 million template. (PDF) [file pone.0202812.s033.pdf]

S22 Table eQTL demographics across the four Roadmap functional affiliations. The numbers refer to the ∼9 million template.

		Strong Enhancer	Weak Enhancer	Active Promoter	Weak Promoter
Adipose	eQTL	836	479	298	658
	Control	1303	1401	457	1257
Epidermal	eQTL	454	376	430	414
	Control	1071	1118	888	902
LCL	eQTL	698	629	704	341
	Control	774	1562	838	588
Whole blood	eQTL	246	174	75	384
	Control	480	567	115	1076
All	eQTL	1507	1758	1726	874
	Control	3436	6081	3685	2355
